# Ligand Ratio Plays a Critical Role in the Design of Optimal Multifunctional Gold Nanoclusters for Targeted Gastric Cancer Therapy

**DOI:** 10.1021/acsnanoscienceau.1c00008

**Published:** 2021-07-16

**Authors:** María Francisca Matus, Sami Malola, Hannu Häkkinen

**Affiliations:** ^†^Department of Physics and ^‡^Department of Chemistry, Nanoscience Center, University of Jyväskylä, FI-40014 Jyväskylä, Finland

**Keywords:** gold nanoclusters, gastric cancer, targeted cancer therapy, biocompatible nanoparticles, drug delivery systems, nanomedicine, peptide−protein interactions, atomistic simulations

## Abstract

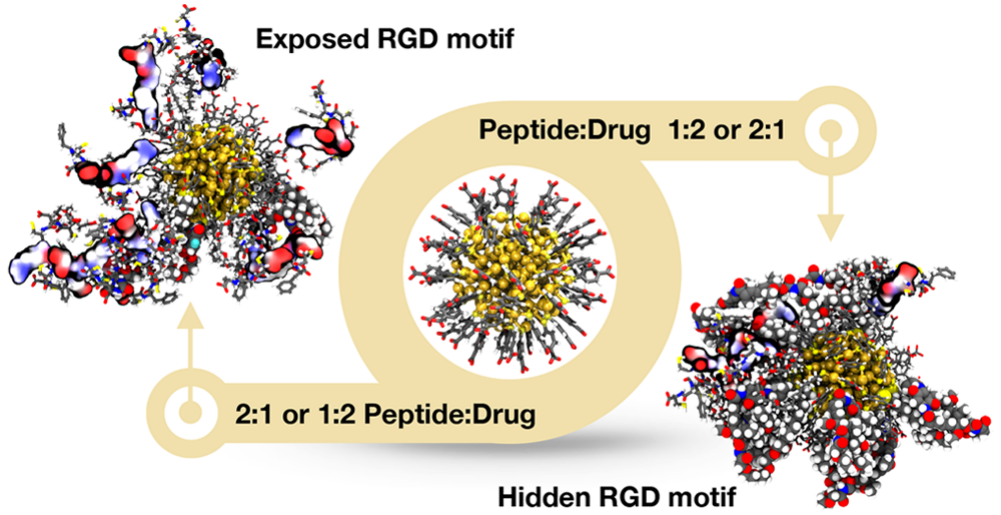

Nanodrug delivery systems (NDDSs) based on water-soluble and atomically precise gold nanoclusters (AuNCs) are under the spotlight due to their great potential in cancer theranostics. Gastric cancer (GC) is one of the most aggressive cancers with a low early diagnosis rate, with drug therapy being the primary means to overcome its increasing incidence. In this work, we designed and characterized a set of 28 targeted nanosystems based on Au_144_(*p*-MBA)_60_ (*p*-MBA = *para*-mercaptobenzoic acid) nanocluster to be potentially employed as combination therapy in GC treatment. The proposed multifunctional AuNCs are functionalized with cytotoxic drugs (5-fluorouracil and epirubicin) or inhibitors of different signaling pathways (phosphatidylinositol 3-kinases (PI3K)/protein kinase B (Akt)/mammalian target of the rapamycin (mTOR), vascular endothelial growth factor (VEGF), and hypoxia-inducible factor (HIF)) and RGD peptides as targeting ligands, and we studied the role of ligand ratio in their optimal structural conformation using peptide–protein docking and all-atom molecular dynamics (MD) simulations. The results reveal that the peptide/drug ratio is a crucial factor influencing the potential targeting ability of the nanosystem. The most convenient features were observed when the peptide amount was favored over the drug in most cases; however, we demonstrated that the system composition and the intermolecular interactions on the ligand shell are crucial for achieving the desired effect. This approach helps guide the experimental stage, providing essential information on the size and composition of the nanosystem at the atomic level for ligand tuning in order to increase the desired properties.

## Introduction

Nanodrug delivery systems (NDDSs) have shown great potential in the diagnosis and therapy of several diseases^1−3^ due to their unique physical and chemical properties, allowing for a controlled and sustained release of drugs. Currently, most NDDSs are focused on cancer theranostics^1,4^ since it remains the major healthcare problem worldwide with increasing incidence.^5^ Gastric cancer (GC), also called stomach cancer, is the third leading cause of cancer death worldwide and the fifth most commonly diagnosed cancer with over 1.0 million new cases in 2018.^5^ Since the early diagnosis rate of GC in most countries is low, the development of new NDDSs represents a valuable tool to improve the treatment of GC.^6−8^

Recent years have seen a surge of atomically precise and ligand-stabilized gold nanoclusters (AuNCs) as one of the most promising materials for cancer nanomedicine.^9,10^ Several studies have demonstrated that the biocompatibility of water-soluble and monodisperse AuNCs can be much improved due to their facile surface modification.^9−11^ For instance, *para*-mercaptobenzoic acid (*p*-MBA)-protected AuNCs can participate in thiolate-for-thiolate ligand exchange (LE) reactions,^12−15^ which is particularly attractive for site-selective modification on the ligand layer and to diversify their surface properties to achieve successful AuNC–biomolecule conjugates.^9,16^ Hence, the precise control of the ligand shell composition makes this class of AuNCs an ideal candidate for the development of targeted NDDSs, which include additional functionalities that allow the therapeutic agent to be selectively delivered to the target site.^17^ These functionalities include antibodies, aptamers, and peptides for biomarker detection,^17,18^ among which peptides stand out for their small size, ease of synthesis and modification, high stability, and good biocompatibility.^19,20^

The use of targeted NDDSs is generally motivated by the expression and/or overexpression of tumor-specific receptors. In this context, αVβ3 integrin, an essential transmembrane receptor for cell adhesion,^21,22^ has been reported as one of the most attractive targets due to its critical role in angiogenesis and metastasis of solid tumors in various cancer subtypes, including GC.^23−25^ This integrin recognizes and binds to the tripeptide motif arginine-glycine-aspartic acid (RGD). Considering the opportunity to design biocompatible ligand-protected AuNCs *via* ligand tuning, here lies an opportunity that needs to be explored in detail.

Computational methods, such as density functional theory (DFT) and molecular dynamics (MD) simulations, have contributed to elucidate structural and physicochemical properties of AuNCs at the atomic level that cannot be explained from a purely experimental perspective.^9,26^ In the design of optimal nanosystems, computational tools, either as a predictive tool or as complementary to experimental work, are indispensable.^9^ Theory–experiment investigations allow for addressing several challenges related to the elucidation of the geometric and electronic structure of the potential nanocarriers, the stability of their ligand shell, and the interaction with the surrounding environment.^26,27^

In this work, we designed a set of targeted nanosystems based on an Au_144_(*p*-MBA)_60_ nanocluster, which has a suitable size (∼1.7 nm metal core diameter) and has been well-characterized before in organic phase with different thiolate ligands,^28,29^ to be potentially employed in GC therapy. The AuNCs are functionalized with cytotoxic drugs (5-fluorouracil and epirubicin) or inhibitors of different signaling pathways (phosphatidylinositol 3-kinases (PI3K)/protein kinase B (Akt)/mammalian target of the rapamycin (mTOR), vascular endothelial growth factor (VEGF), and hypoxia-inducible factor (HIF)) and RGD peptides as targeting ligands, and we explored the role of ligand ratio in their optimal structural conformation using peptide–protein docking and MD simulations. This approach serves as a predictive tool for the experimental phase, providing essential information on the size and composition of the nanosystem at the atomic level, and facilitates the decision-making process to determine which features of the nanosystem can be adapted to enhance the desired properties.

## Results and Discussion

### Selection of Best Peptide Candidates for Targeting

**Figure 1:**
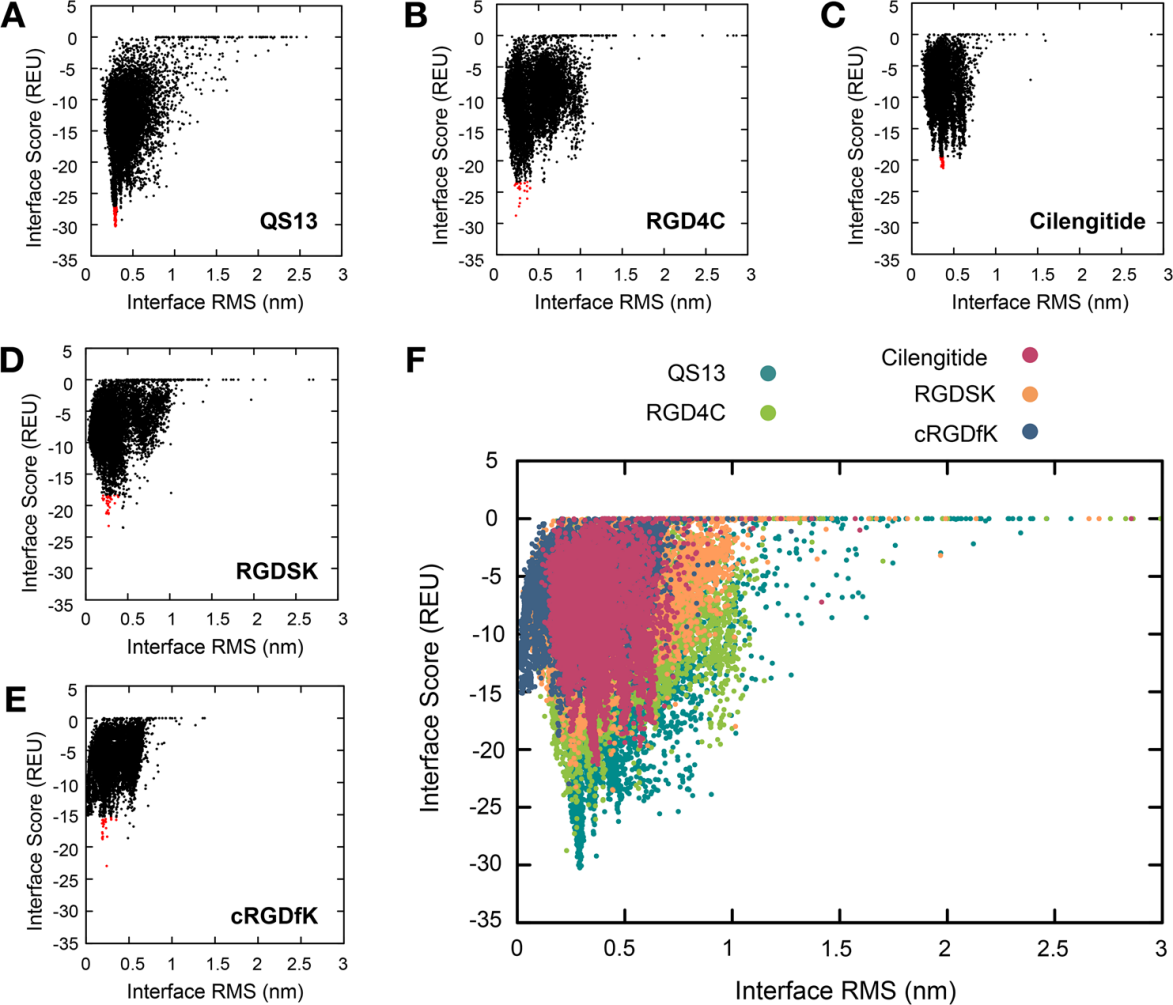
Docking predictions for RGD peptides−αVβ3 integrin complex. Interface score versus interface root mean square (RMS) scatter plot for 10 000 models of (A) QS13, (B) RGD4C, (C) cilengitide, (D) RGDSK, and (E) cRGDfK peptides complexed with αVβ3 integrin. Red points represent the best quality predictions. (F) Superposition of the same energy landscapes for better comparison. REU, Rosetta energy units.

**Figure 2:**
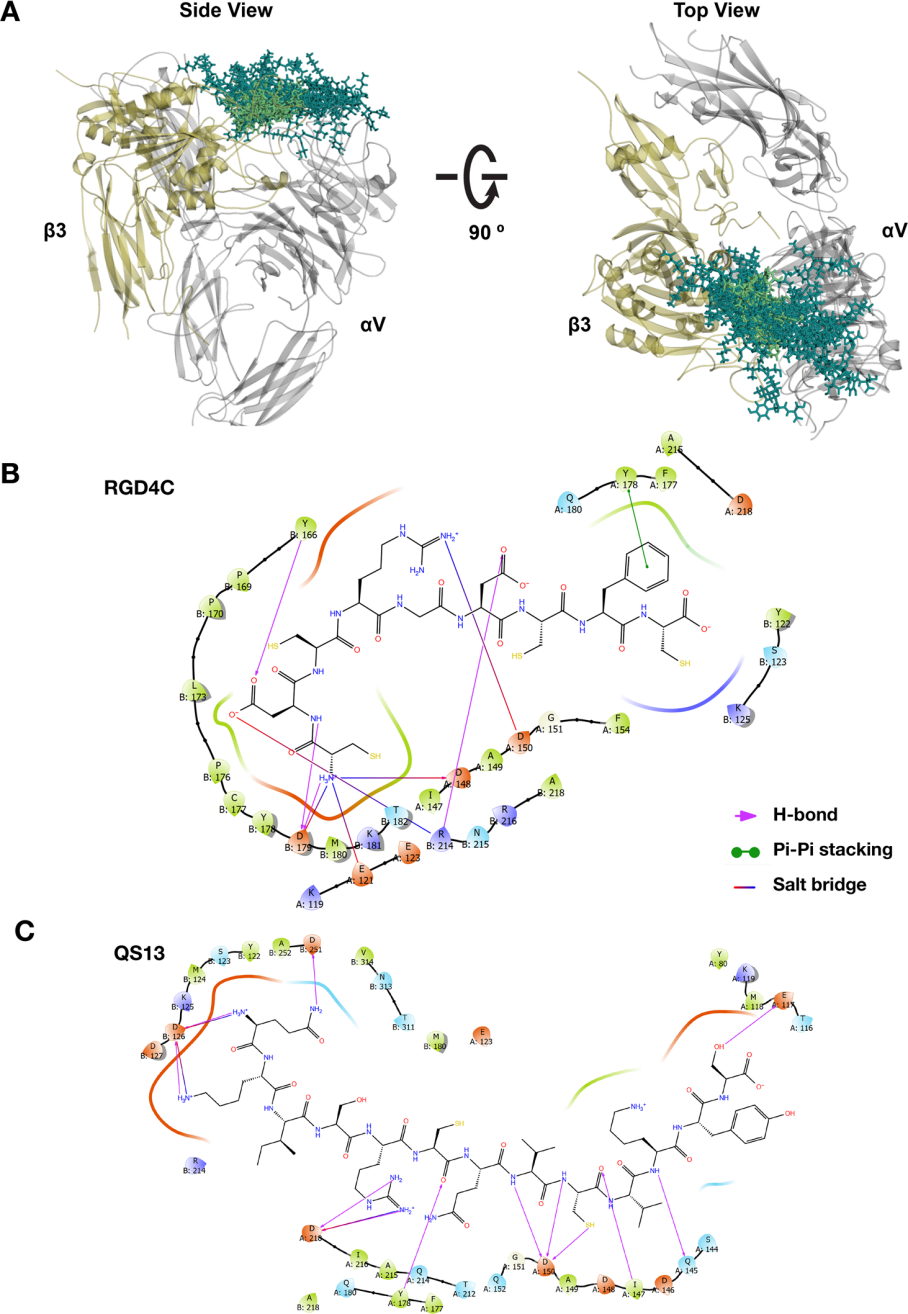
Binding modes of the best peptides candidates in the αVβ3 binding site. (A) Superimposition of the 10 lowest energy poses obtained for QS13 (dark cyan sticks) and RGD4C (green sticks) in the αVβ3 binding site (αV subunit: gray cartoon; β3 subunit: yellow cartoon). 2D interaction diagrams of (B) RGD4C and (C) QS13 peptides. QS13 sequence: QKISRCQVCVKYS. RGD4C sequence: CDCRGDCFC. The αVβ3 residues are colored as follows: charged positive, blue; charged negative, red; hydrophobic, green; polar, cyan; glycine, white.

We employed the RGD-strategy by using a set of linear and cyclic peptides that have shown promising results as integrin-specific ligands in different formulations,^30^ such as cyclo(RGDf-NMeV) (cilengitide),^31^ cyclo(RGDfK),^32^ RGDSK,^33^ CDCRGDCFC (RGD4C),^34^ and QKISRCQVCVKYS (QS13).^35^ We first analyzed the interactions of the peptides with the cancer cell receptor through peptide–protein docking using the crystal structure of the extracellular segment of αVβ3 in complex with cilengitide as a reference (PDB ID: 1L5G).^36^Figure 1 shows the docking scores of all 10 000 models generated for each case. Both cyclic and linear peptides show a wide energy funnel. However, QS13 (Figure 1A) and RGD4C (Figure 1B) show a deeper funnel with the best degree of convergence (where the lower energy values correspond to lower interface root mean square (RMS) values), suggesting that these interfaces are less flexible than the other three (Figure 1C–F). The docking results were then ranked according to the binding energy calculated by separating and repacking both subunits present in each complex. QS13– and RGD4C–integrin complexes showed the best binding affinity with −32.33 ± 7.4 and −20.30 ± 4.7 REU (Rosetta energy units), respectively (Table S1). The 10 best poses (lowest binding energy) obtained for RGD4C and QS13 bind at the top of the β-propeller domain, making contacts with both αV and β3 subunits (Figure 2A), which correspond to the well-known RGD binding site.^36^ The RGD4C peptide forms hydrogen-bond interactions and salt bridges with some critical residues in the binding site such as D179, D150, and R214 through the side chain or backbone amide of ^2^D, ^4^R, and ^6^D (Figure 2B). Additional contacts involve a π–π stacking between Y178 and ^8^F and a hydrogen bond between Y166 and the carboxylate group of ^2^D. As previously reported,^35^ although the QS13 peptide does not have an RGD motif, we observed that its binding site on αVβ3 overlaps the RGD binding site. The ^5^R^6^C^7^Q^8^V^9^C portion of the peptide stabilizes the interaction with the receptor through a hydrogen-bond network formed between either the side chains or the backbone amide of this sequence motif and some key residues like D150, Y178, D218, I147, and Q145 (Figure 2C). ^2^K also seems to be crucial for the binding mode due to the formation of hydrogen bonds and salt bridges with D126, allowing for the interaction with the β3 subunit together with the hydrogen-bond interaction between ^1^Q and D251. Interestingly, QS13 has been shown to inhibit the focal adhesion kinase (FAK)/PI3K/Akt pathway,^35^ which might confer some additional pharmacological action to the nanosystem for the proposed targeted cancer therapy. On the basis of the predicted potential mechanism of binding, we selected both QS13 and RGD4C peptides as the best candidates for designing the proposed AuNC-based targeted drug delivery system.

### Structural Characterization of Multifunctional Gold Nanoclusters

**Figure 3:**
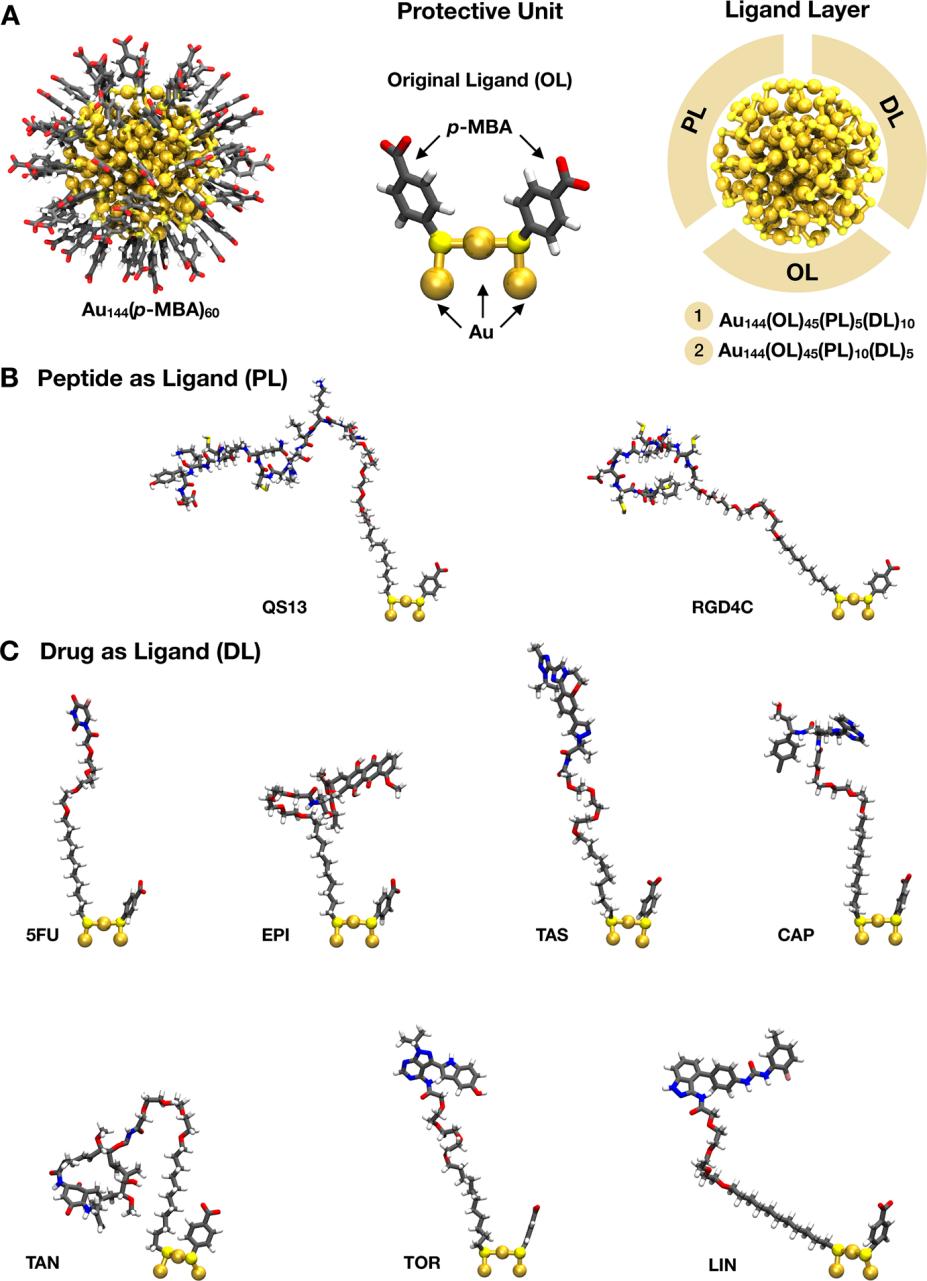
Schematic representation of the functionalization procedure of Au_144_(*p*-MBA)_60_ nanocluster. (A) Reference structure of the Au_144_(*p*-MBA)_60_ nanocluster (*p*-MBA denotes *para*-mercaptobenzoic acid) highlighting the original protective unit containing two *p*-MBA ligands that will be modified during the functionalization procedure to obtain the multifunctional gold nanoclusters using two peptide/drug ratios (1:2 and 2:1). The new ligand layer of Au_144_ nanocluster is composed of 45 original *p*-MBA ligands (OL) and 15 new incoming ligands integrated by (B) the targeting peptides (PL) and (C) chemo-drugs or signaling pathway inhibitors (DL). Peptides and drugs are covalently attached to the protective unit through a thiolate polyethylene-glycol (PEG) linker, which acts as a spacer arm. 5FU, EPI, TAS, CAP, TAN, TOR, and LIN denote 5-fluorouracil, epirubicin, taselisib, capivasertib, tanespimycin, torkinib, and linifanib, respectively. Gold atoms are depicted as spheres and different ligands as sticks using the conventional color code: gold, golden yellow; sulfur, yellow, carbon, gray; oxygen, red; nitrogen, blue; hydrogen, white; fluoride, pink; chloride, dark gray.

Au_144_(*p*-MBA)_60_ nanocluster (Figure 3A) used as a drug carrier comprises an appropriate balance between size (∼1.7 nm metal core diameter) and number of surface sites to be occupied by new incoming ligands.^28^ We built various models of multifunctional Au_144_ nanoclusters by using different ligand ratios on the basis of the experimental evidence of LE rate^13^ (Figure 3A) to study the energetic contribution of each combination. Thiolate-polyethylene glycol (PEG) was used as a spacer arm^37,38^ to conjugate the targeting ligands (Figure 3B) or the anticancer drugs (Figure 3C) replicating the EDC/NHS coupling reaction approach.^39^ This method has been reported before for the conjugation of doxorubicin to PEGylated gold nanoparticles with a high reaction efficiency.^38^ We explored the functionalization with the current first-line therapeutics in systemic chemotherapy for GC: 5-fluorouracil (5FU) and epirubicin (EPI).^40,41^ Also, different inhibitors of signaling pathways that have been identified as the molecular mechanism underlying cell survival under cytotoxic pressure in GC were included:^42−45^ linifanib (LIN; VEGF inhibitor),^46^ tanespimycin (TAN; HIF inhibitor),^43^ taselisib (TAS; PI3K inhibitor),^47^ torkinib (TOR; PI3K/Akt/mTOR inhibitor),^48^ and capivasertib (CAP; Akt inhibitor),^49^ with the aim to predict the successful association for a potential targeted drug combination therapy.

**Figure 4:**
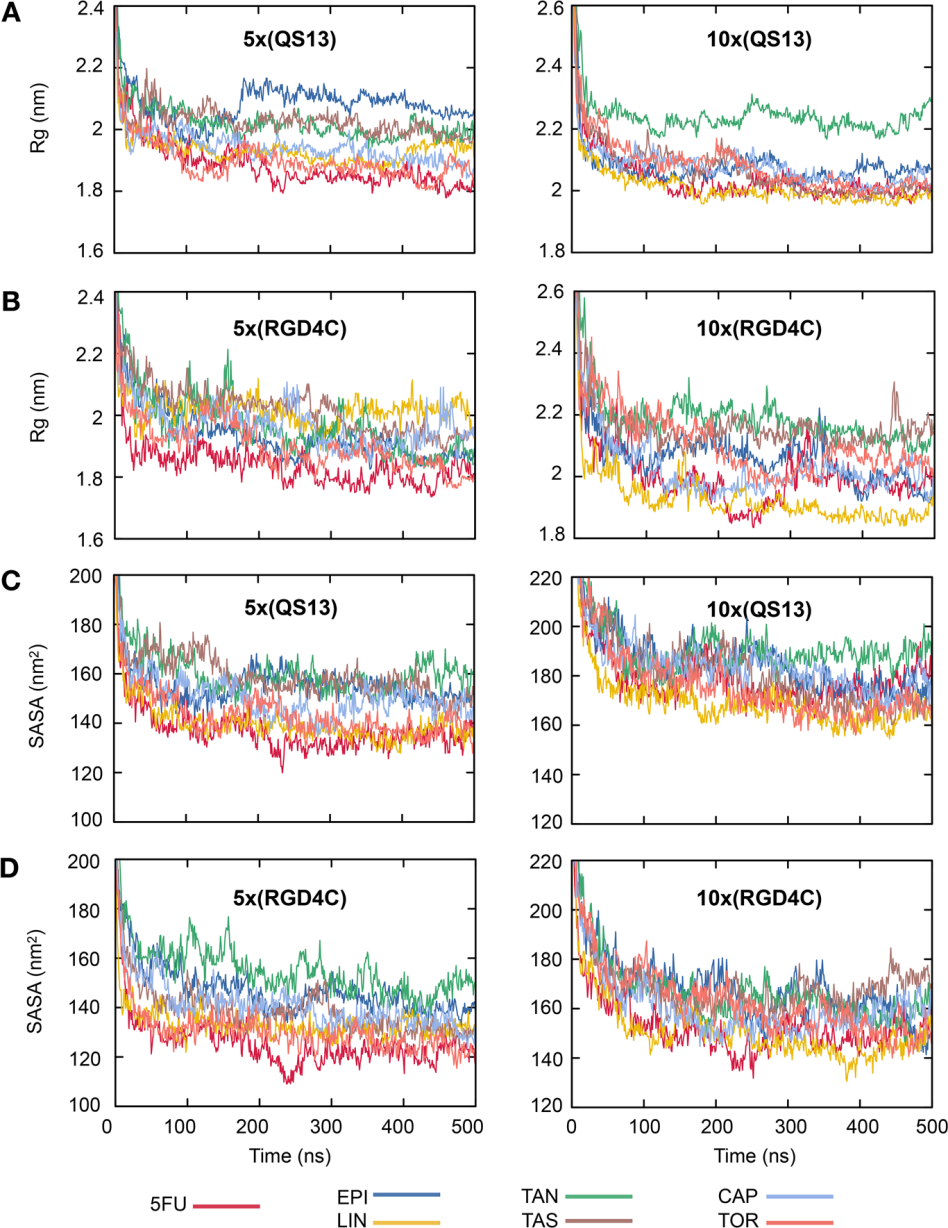
Spatial distribution and hydrophilicity of multifunctional gold nanoclusters. Radii of gyration (Rg) for functionalized nanoclusters using (A) QS13 or (B) RGD4C peptide as a targeting ligand and solvent accessible surface area (SASA) for functionalized nanoclusters with (C) QS13 or (D) RGD4C peptides as a function of simulated time. Left panels: peptide/drug ratio at 1:2. Right panels: peptide/drug ratio at 2:1. Average Rg and SASA for the reference structure Au_144_(*p*-MBA)_60_ (*p*-MBA denotes *para*-mercaptobezoic acid) is 1.17 ± 0.002 nm and 56.85 ± 0.73 nm^2^, respectively. 5FU, EPI, LIN, TAN, TAS, CAP, and TOR denote 5-fluorouracil, epirubicin, linifanib, tanespimycin, taselisib, capivasertib, and torkinib, respectively.

Parts A and B of Figure 4 show the fluctuation of the radius of gyration (Rg) during 500 ns simulation time for each drug–conjugated AuNC containing different QS13 (Figure 4A) or RGD4C (Figure 4B) peptide ratios. Radii of gyration take on a steady value after 30 ns of simulation, which indicates that all the modeled structures are fully equilibrated in our simulations. The increase in size is nearly twice compared to the original Au_144_(*p*-MBA)_60_ structure, resulting in a diameter of ∼4 nm for most cases (Tables S2 and S3). The observed ultrasmall size of all the functionalized NCs is smaller than the glomerular filtration threshold (<6 nm diameter),^50,51^ which may favor the efficient renal clearance after treatment and consequently minimizing the potential side effects due to their long-term accumulation in healthy tissues/organs.^52,53^ Among the AuNCs functionalized with QS13, we observed the largest sizes when they are conjugated with EPI (Rg: 2.07 ± 0.05 nm) and TAN (Rg: 2.23 ± 0.03 nm) in peptide/drug ratios of 1:2 and 2:1, respectively (Figure 4A), while for RGD4C-functionalized AuNCs, the largest sizes were obtained with TAS (Rg: 2.01 ± 0.06 nm) and TAN (2.17 ± 0.05 nm) in peptide/drug ratios of 1:2 and 2:1, respectively (Figure 4B). These results suggest that the AuNC size depends not only on how big or rigid the drug molecule is but also on the intermolecular interaction between the different units in the nanosystem: core, cargo, and targeting portion.

**Figure 5:**
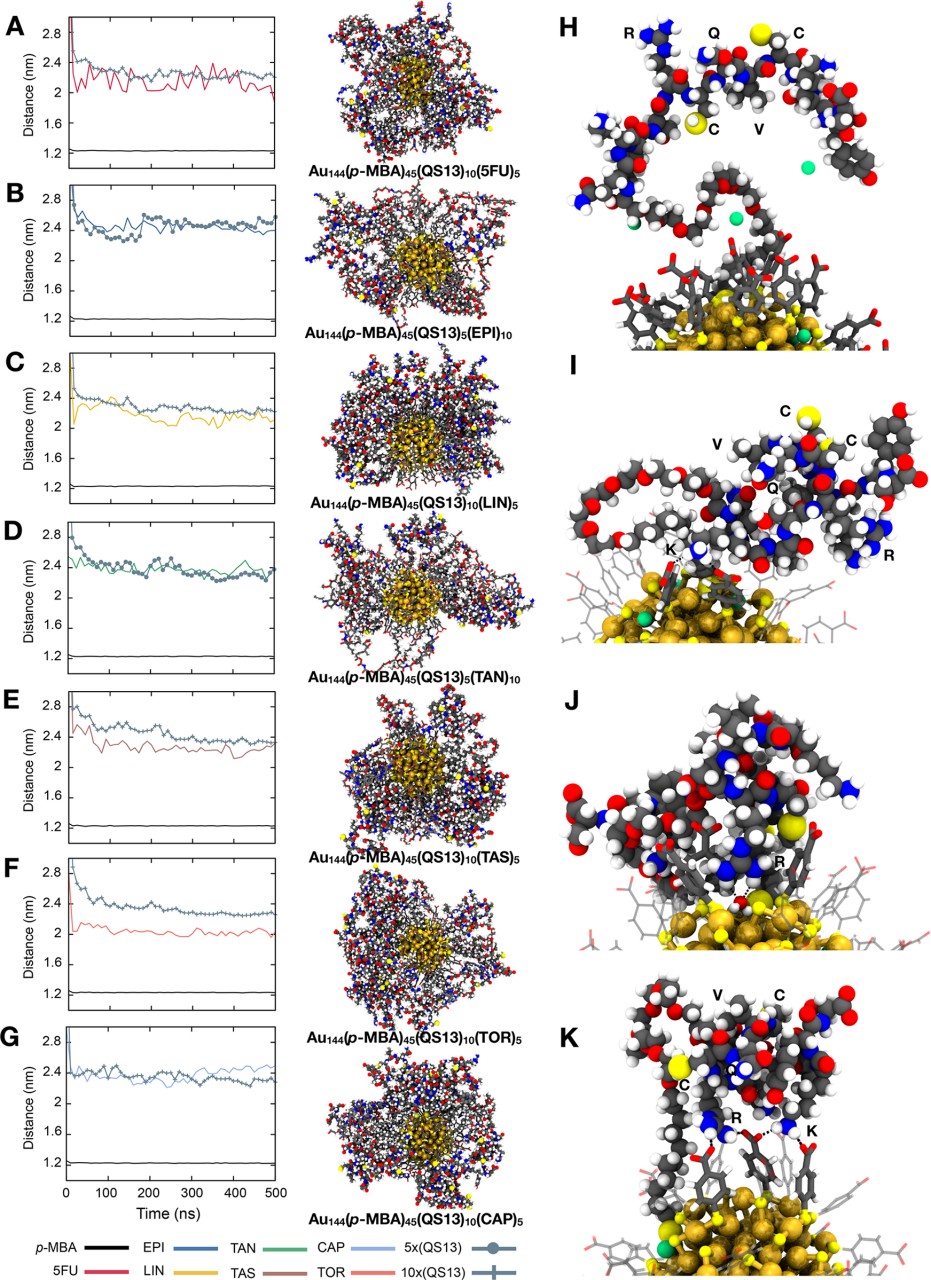
Distribution of the RCQVC motif of QS13 peptide on the ligand layer of most promising multifunctional gold nanoclusters. Average distance between the gold core and the different components in the ligand layer (*para*-mercaptobenzoic acid, drug, and targeting motif of peptide) when the nanocluster is conjugated with (A) 5-fluorouracil (5FU) (B) epirubicin (EPI), (C) linifanib (LIN), (D) tanespimycin (TAN), (E) taselisib (TAS), (F) torkinib (TOR), or (G) capivasertib (CAP). Right panels show a snapshot of the whole nanosystem after 500 ns of simulation when the optimal peptide/drug ratio is used. Gold core is depicted as spheres, drugs as sticks, and peptides as balls. Representative cases of RCQVC motif orientation are also illustrated when it is (H) completely or (I) relatively exposed to the solvent or when it is (J and K) interacting with the other components of the nanosystem (drugs are omitted for clarity). Hydrogen-bond interactions are depicted as black dashed lines. Color code: gold, golden yellow; sulfur, yellow, carbon, gray; oxygen, red; nitrogen, blue; hydrogen, white; fluoride, pink; chloride, dark gray; sodium, green.

**Figure 6:**
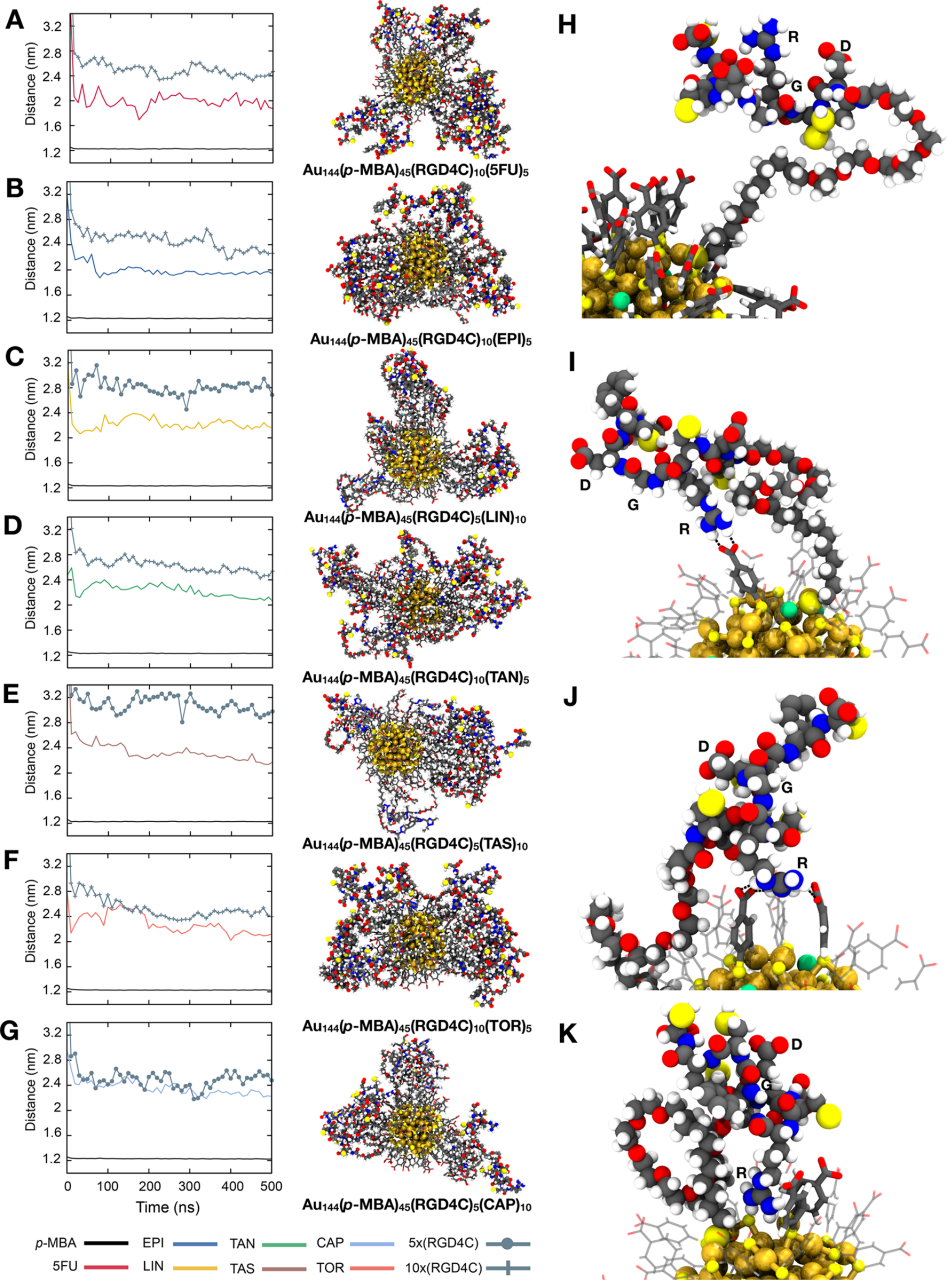
Distribution of the RGD motif of RGD4C peptide on the ligand layer of most promising multifunctional gold nanoclusters. Average distance between the gold core and the different components in the ligand layer (*para*-mercaptobenzoic acid, drug, and targeting motif of peptide) when the nanocluster is conjugated with (A) 5-fluorouracil (5FU) (B) epirubicin (EPI), (C) linifanib (LIN), (D) tanespimycin (TAN), (E) taselisib (TAS), (F) torkinib (TOR), or (G) capivasertib (CAP). Right panels show a snapshot of the whole nanosystem after 500 ns of simulation when the optimal peptide/drug ratio is used. Gold core is depicted as spheres, drugs as sticks, and peptides as balls. Representative cases of RGD motif orientation are also illustrated when it is (H) completely or (I) relatively exposed to the solvent or when it is (J and K) making contact with the other components of the nanosystem (drugs are omitted for clarity). Hydrogen-bond interactions are depicted as black dashed lines. Color code: gold, golden yellow; sulfur, yellow, carbon, gray; oxygen, red; nitrogen, blue; hydrogen, white; fluoride, pink; chloride, dark gray; sodium, green.

On the contrary, when the solvent-accessible surface area (SASA) is compared between the different functionalized AuNCs, we observed a similar behavior either using QS13 (Figure 4C) or RGD4C (Figure 4D) as the targeting ligand. The decrease in SASA during the first 30 ns is due to the stabilization of the system where the peptides and PEG chains folding process takes place. After 30 ns, the SASA is constant for all different systems, ranging from 135.69 to 159.02 and from 167.69 to 189.17 or from 124.10 to 153.77 and from 148.76 to 166.53 nm^2^ for QS13- or RGD4C-functionalized systems in peptide/drug ratios of 1:2 and 2:1, respectively (Tables S2 and S3). We furthermore calculated the SASA by component: *p*-MBA ligands, PEG-drug, and PEG-peptide conjugates. The results show that the total SASA of each functionalized system is governed by the hydrophilicity of the peptides located on the outermost surface of the ligand layer (Tables S2 and S3, Figures 5 and 6). The linkers seem to have an essential dual function on the nanosystem conformation. On the one hand, they increase the solubility of the nanosystem due to the PEG hydrophilicity, and on the other hand, they favor the accessibility of the peptides to the solvent, which consequently will favor the exposure of the targeting portion.

### Ligand Ratio Dependency for Targeting Ability of Multifunctional Gold Nanoclusters

Besides the size and characterization of each component distribution within the nanosystem, it is imperative to analyze the RGD motif arrangement in each formulation and how this is disrupted/favored by the different ligand ratios at the AuNC surface. Thus, the best formulations will be those in which the targeting portion is far enough from the other ligands and therefore is free to interact with the cancer cell receptor. For this purpose, we analyzed the average distance of the RCQVC portion of QS13 (Figure 5) and the RGD motif of the RGD4C peptide (Figure 6) with respect to the Au_144_ core as a function of simulated time. This parameter was also evaluated for the *p*-MBA ligands and the drug conjugated in each nanosystem. We observed that the potential targeting ability of the proposed nanosystems highly depends on the ligand ratio used for their functionalization. For instance, as shown in Figure 5, most of the systems exhibit the desired peptide orientation when QS13 is used in the functionalization with the peptide/drug ratio 2:1, including 5FU- (Figure 5A), LIN- (Figure 5C), TAS- (Figure 5E), TOR- (Figure 5F), and CAP-conjugated (Figure 5G) nanoclusters. The highest difference between peptide–gold core (2.33 ± 0.08 nm) and drug–gold core (2.04 ± 0.05 nm) distances was observed when the system is conjugated with TOR, while the rest of these systems show a difference of only ∼0.12 nm (Table S4). We suggest that this is highly related both to the size and hydrophobicity of the drug (calculated octanol/water partition coefficient of 9.71 for PEG-TOR; see Table S6), which creates an intermediate hydrophobic region between *p*-MBA and peptide ligands, therefore, keeping the RCQVC motif positioned far enough from the inner part. By contrast, when the gold nanocluster is conjugated with EPI (Figure 5B) or TAN (Figure 5D), the most favorable peptide/drug ratio is 1:2. In all these systems, the RCQVC portion was mainly observed facing out, leading to better exposure of the side chains, as illustrated in Figure 5H. Another representative case is shown in Figure 5I, in which the RCQVC portion is relatively free on the surface, but some interactions occur between other residues of the peptide and the *p*-MBA ligands. For example, the hydrogen bonds formed between the side chain of ^2^K and the deprotonated *p*-MBA cause the peptide to fold slightly inward, but the RCQVC portion is still accessible for the cell receptor. It is worth noting that ^2^K also plays a pivotal role in the interaction with the αVβ3 integrin (as discussed before and shown in Figure 2C); therefore, when this kind of interaction is established, it might affect the targeting ability of the nanosystem even if the RCQVC portion is relatively free. A clear sign of the dynamic behavior of these systems is exhibited during the last 200 ns for CAP- (Figure 5G) and TAN-conjugated (Figure 5D) nanosystems, where the drug is observed in the outermost layer. The situation is worsened when the peptide/drug ratio is altered in the formulation, with the drug being more exposed than the peptide during all the simulated time (Figure S6). A similar result was found when TAS was favored over QS13 (Figure S6), which serves as a serious obstacle to the targeting process. As an example, when ^5^R (from the RCQVC motif) establishes interactions toward the gold core with the inner ligands, we observed two main undesired conformations: (1) forming hydrogen bond interactions with some water molecules located at protective unit level (Figure 5J) and (2) making multiple contacts (hydrogen-bond networks) together with some lysines and the carboxylate groups of *p*-MBA (Figure 5K), causing that the targeting portion to be somehow hidden, and therefore, it would make the receptor recognition mechanism more difficult.

A similar finding was observed for RGD4C-functionalized nanoclusters. When the peptide/drug ratio was 2:1, four out of seven systems showed the desired orientation, such as 5FU- (Figure 6A), EPI- (Figure 6B), TAN- (Figure 6D), and TOR-conjugated (Figure 6F) nanoclusters. For those nanosystems containing 5FU and EPI, we could observe that they both exhibited convenient features for targeting regardless of the ligand ratio employed (Figure 6 and Figure S7); however, a more marked difference between the drug and peptide components with respect to the gold core (∼0.5 versus ∼0.2 nm) was found when the peptide amount was favored over the drug (Table S5). TAN-conjugated nanosystems also showed an appropriate RGD motif orientation during the first half of the simulation, either using peptide/drug ratios 1:2 or 2:1 (Figure 6D and Figure S7). However, after 250 ns, the peptide moved closer to the drug layer when TAN was favored in the formulation, while its counterpart remained more stable during all the simulated time with a difference between the peptide–gold core and drug–gold core distances of around 0.4 nm (Table S5). On the contrary, the peptide/drug ratio at 1:2 appeared to be a better option when the system was conjugated with LIN (Figure 6C), TAS (Figure 6E), or CAP (Figure 6G), among which LIN- and TAS-conjugated nanosystems exhibit the clearest distinction of the three levels on the ligand layer. The RGD motif orientation for the most favorable formulations is represented in Figure 6H. On the contrary, for those unfavorable combinations, we observed three representative cases: (1) ^4^R (from the RGD motif) interacts with one *p*-MBA ligand through hydrogen bonding, while ^5^G and ^6^D are still relatively accessible at the surface (Figure 6I); (2) at least two *p*-MBA ligands contribute to a more stable interaction with the side chain of ^4^R, but ^6^D and ^8^F are located away from the gold core (Figure 6J), which might provide a chance to interact with the receptor and favor the outward folding; (3) ^4^R is located close to the inner core and all the RGD motif residues are facing inward as well (Figure 6K), which represents the most undesirable condition.

Our findings show that the general tendency is to add more peptide than drug into the nanosystem to achieve the best formulations, either using QS13 or RGD4C. However, it strongly depends on the drug employed in each case and the intermolecular interactions that take place in the ligand layer.

We observed a more evident three-level ligand layer in most RGD4C-functionalized nanosystems than QS13-functionalized ones, with the drugs located in the middle part (Figures 5 and 6). The drug layer is, in a sense, protected by the outer peptide layer, which is an excellent advantage for the potential route that the nanosystems will experience and demonstrates the importance of employing different lengths of PEG as a linker with the gold core, which at the same time, confers a higher stability and reduction of immunogenicity, to name a few.^54^ In fact, this factor could be adjusted for those formulations containing the QS13 peptide in order to obtain a more exposed targeting portion. For instance, employing a PEG linker with a little longer ethylene-glycol chain might help achieve this or even testing an additional peptide/drug ratio, like 3:1 or 4:1. However, by using the latter strategy, there is the risk of significantly limiting the effectiveness of the nanosystem since the required drug loading for the anticancer effect may not be achieved.

This first approach about the optimal ligand ratio based on the LE strategy is also helpful for controlling the ligand density on the AuNCs surface. It has been reported that the number of targeting ligands needs to be precisely controlled in NDDSs to accomplish the desired effect.^55,56^ LE reactions offer an excellent opportunity for controlling the size and surface composition of the proposed nanosystem, providing crucial information for guidance on experimental design.

### Distribution of Sodium Counterions in the Ligand Layer of Multifunctional Nanoclusters

**Figure 7:**
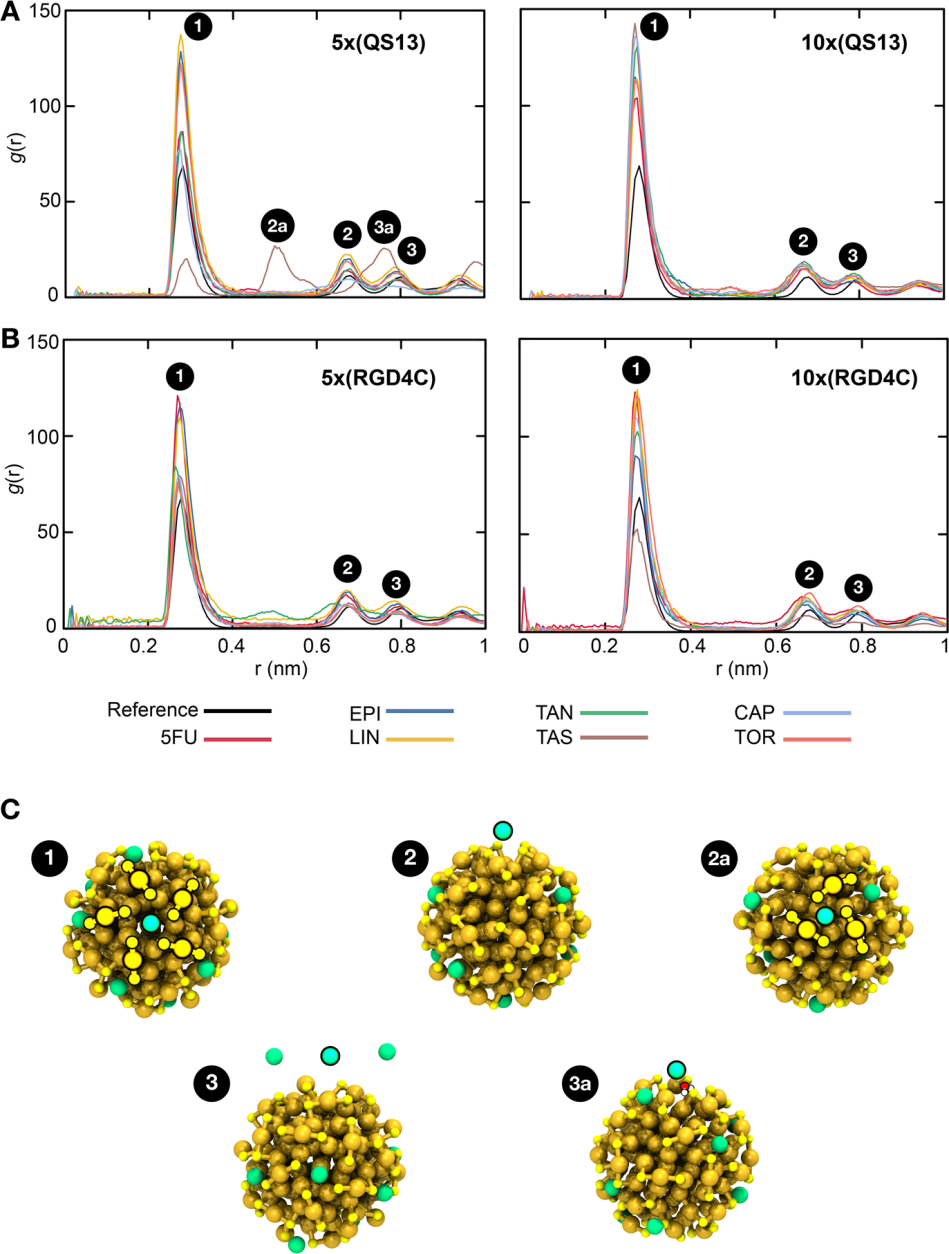
Interaction of sodium counterions with the inner core of multifunctional nanoclusters. Radial distribution function (RDF) between sodium ions and sulfur atoms at the RS-Au-SR units (where R = *para*-mercaptobenzoic acid, peptides, or drugs) in the protective layer of Au_144_ nanoclusters functionalized with (A) QS13 or (B) RGD4C peptides, and (C) representative snapshots of the observed interactions. Left panels: peptide/drug ratio at 1:2. Right panels: peptide/drug ratio at 2:1. 5FU, EPI, LIN, TAN, TAS, CAP, and TOR denote 5-fluorouracil, epirubicin, linifanib, tanespimycin, taselisib, capivasertib, and torkinib, respectively. Gold core and sodium cations are depicted as spheres and water molecules as balls and sticks. Color code: gold, golden yellow; sulfur, yellow, oxygen, red; hydrogen, white; sodium, green.

We also investigated the effect on the diffusion of the counterions for each functionalized nanocluster. The radial distribution function (RDF) between sodium counterions (Na^+^) and the gold core for each system is shown in Figure 7. It can be found that all curves of QS13- (Figure 7A) or RGD4C-functionalized (Figure 7B) nanoclusters show similar features, and they showed a uniform distribution of Na^+^ cations within them. The first and main peak appears at about 0.27 nm, and its intensity increases with the functionalization process. At this distance, the Na^+^ cation interacts with the gold core *via* coordination with five protective units with the Na^+^ located at the center (Figure 7C) and is stable during the simulation time. The increase of the intensity in the first peak is more prominent for those QS13-functionalized systems employing a peptide/drug ratio of 2:1 (Figure 7A, right panel), and it can be attributed to the more hydrophilic character of those systems (calculated logP values of QS13 and RGD4C peptides are −0.74 and 0.49, respectively),^57^ which allows Na^+^ cations and water molecules go inside and stabilize the coordination and interactions with the inner portion of the nanosystem. The second and third minor peaks appear at around 0.67 and 0.79 nm, showing the same shape in all different systems and only a slight height change. These peaks have been identified as the interactions between Na^+^ and sulfur atoms in the protective units (which have a negative partial charge), but when Na^+^ cations are located a little further from the innermost part or even at *p*-MBA ligand level (Figure 7C). An exceptional situation was observed for the QS13-TAS-functionalized nanocluster (peptide/drug ratio 1:2), where the second and third peaks appeared at around 0.5 and 0.75 nm (Figure 7A). The second peak is represented by the coordination of a Na^+^ cation with three protective units (Figure 7C), and the third peak can be assigned to a solvent-shared ion pair, in which a water molecule is located in between the Na^+^ ion and the gold core (Figure 7C). We previously reported a similar behavior between Au_25_(PET)_18_ (PET = phenylethylthiolate) nanocluster and cesium cation (Cs^+^) in the gas phase,^58^ where the Cs^+^ was observed interacting with the ligand layer and then located at the center of three protective units.

## Conclusions

In this work, we have designed and characterized, at the atomic level, the structures of a set of multifunctional nanosystems for GC therapy based on *p*-MBA-protected gold nanoclusters and RGD peptides for the targeted delivery of cytotoxic drugs or signaling pathway inhibitors. We observed that the potential targeting ability and the consequent therapeutic effect are governed by the ligand ratio employed in the formulation and their distribution in the ligand layer. It could be assumed *a priori* that, the more targeting ligand we add to the nanosystem, the more effective to target the cancer cell. However, we demonstrated that the system composition and the intermolecular interactions on the ligand shell are crucial for achieving the appropriate balance between the targeting ability and drug loading.

We studied 28 nanosystems containing different proportions of the targeting ligand and the therapeutic cargo. All of them exhibited suitable properties in terms of biocompatibility and potential efficient renal clearance.^50^ However, the main differences were found in their components distribution. Although they showed a dynamic behavior, as observed from the MD simulations, we were able to identify some representative situations and discriminate the favorable and unfavorable formulations. The most convenient features were observed when the peptide amount was favored over the drug, either with RGD4C or QS13 as the targeting ligand, allowing the targeting motif to be adequately exposed to the solvent and facilitating the recognition process. One remarkable feature of our proposed nanosystem is that the functionalization phase can be extended to target other types of cancer only by changing the therapeutic cargo. Our model is relevant as it allows a higher atomic-level control of the nanocarrier than what can be achieved with the colloidal noble metal nanoparticles.^9^ It can also be applied for a series of larger water-soluble and monodisperse AuNCs that have shown biocompatible properties such as low toxicity and high solubility.^59^

There is extensive evidence that targeted drug delivery holds the key to the success of most anticancer therapies,^60^ and basic research of the composition of these systems and their efficacy in specific pathologies is imperative. Therefore, we believe that the protocol presented here serves as a preliminary screening to guide the experimental phase, facilitating decision-making during the development phase, and it represents an essential tool for the rational design of AuNCs-based nanocarriers. However, the definition of general guidelines for applying in different classes of hybrid nanosystems is not possible. Different authors have provided guidelines regarding ligand density on the surface of targeted nanoparticles for improved delivery strategies.^55,56^ Nevertheless, our results suggest that the biofunctionalization strategies for NDDSs must be made case-by-case since the physicochemical properties and surface chemistry of nanocarriers are governed by several factors, including the composition of the ligand layer and the interactions between the different structural components.

In this work, we have only considered the impact of ligand ratio and its distribution on the potential targeting functionality of the nanosystem, not focusing on the surface coverage of AuNCs with biomolecules, called “protein corona” (PC). It has been demonstrated that PC directly influences the surface chemistry of NDDSs and, consequently, has a strong implication on their targeting abilities.^61^ Thus, detailed atomic-level modeling of PC formation would be necessary for further investigations to predict the potential discrepancies between the *in vivo* and *in vitro* studies associated with this phenomenon, which are significant challenges to face for most nanomaterials.^62^ Further studies also include the characterization at the atomistic scale of the nanosystem–cancer cell receptor interactions to gain insight into the dynamic behavior and possible structural changes of this complex under realistic conditions, as well as the synthesis and characterization of the most promising formulations to be tested experimentally.

## Methods

In this work, we designed and characterized the structural features of multifunctional targeted gold nanoclusters through all-atom MD simulations. As a first step, we describe the method used to select the best candidates as targeting ligands. Next, we detail the parametrization procedure for the different peptides and drugs that constitute the ligand layer of the gold nanoclusters, followed by the construction of the atomistic models. Finally, we provide the setup protocol for MD simulations of the resulting functionalized nanosystems.

### Peptide–Protein Docking

The 3D models of short RGD peptides (<10 aas) were built using UCSF Chimera software,^63^ and the I-TASSER server^64^ was used to obtain the model of QS13 peptide (13 aas). The protonation states at pH 7.4 were determined using PROPKA 3.0^65^ for each peptide. The crystal structure of the extracellular segment of integrin αVβ3 in complex with Cilengitide (PDB ID: 1L5G)^36^ was used as reference to perform the peptide–protein docking with Rosetta FlexPepDock^66^ using the Refinement protocol.^67^ The cocrystallized structure of cilengitide was taken off from its receptor and redocked into the same binding pocket (Figure S1). For each complex, 10 000 conformations were generated and ranked according to their interface score and binding energy. The peptide–protein interactions were analyzed with Maestro 12.1 software.^68^ Finally, the two best peptide candidates were then selected to be included in the nanosystem.

### Parameterization of Linker-Drugs and Linker-Peptides

The force-field parameters for Au_144_(*p*-MBA)_60_ were described in a previous paper.^69^ However, it was necessary to parametrize the new protective unit that originates after the LE reaction that we needed to replicate for these systems.

First, we conjugated each peptide (QS13 and RGD4C) and drug (5FU, EPI, LIN, TAN, TAS, CAP, TOR) to a long (HS–C_11_–(EG)_6_–OCH_2_–COOH) or short (HS–C_11_–(EG)_3_–OCH_2_–COOH) PEG thiol linker, respectively, under the principle of EDC/NHS conjugation-based method^39^ (Figure 3). Then, we constructed a model of the new protective unit, replacing one of the original *p*-MBA ligands with the above conjugates. Charges were derived for a model system consisting of two gold atoms connected to two ligands *via* a sulfur atom, that is, Au_2_(*p*-MBA)_1_(X)_1_ (where X is PEG-drug or PEG-peptide) in order to describe the rectangular protective unit present in Au_144_ NCs, as previously published.^69^ The force-field parameters for all the different PEG-drugs and PEG-peptides were obtained using suitable available parameters of the Amber99sb-ildn^70^ force field. The partial charges were optimized following the RESP charge fitting procedure recommended for Amber.^71^ The geometry optimization and ESP calculations according to the Merz–Singh–Kollman scheme^72,73^ were performed with Gaussian09^74^ at a B3LYP/LANL2DZ/W06 level of theory.^75^ Atomic charges were fitted to the obtained potential in a two-stage RESP fit procedure with Ambertools12,^76^ constraining the charges of gold to zero and keeping the same *p*-MBA charges reported previously for the deprotonated form.^69^ After that, we obtained the GROMACS topologies for each new conjugate using ACPYPE code.^77^

### Models of Multifunctional Au_144_ Nanoclusters

After their parametrization, the PEG-peptide and PEG-drug structures were assembled into an Au_144_ gold core using different ligand ratios on the basis of the experimental evidence of LE rate.^13^ The peptide/drug ratio was 1:2 or 2:1, resulting in 28 different multifunctional gold nanoclusters containing 45 original *p*-MBA ligands and 15 new incoming ligands (Figures S2–S5).

The basis for all multifunctional model structures was the Au_144_(*p*-MBA)_60_ cluster, which was first built on the basis of the predicted^29^ and later observed^28^ clusters with similar molecular composition but protected by other thiolates than *p*-MBA. The created model cluster of Au_144_(*p*-MBA)_60_ was optimized using DFT as implemented in GPAW software^78^ together with local density approximation (LDA) xc-functional^79^ and real space grid with 0.2 Å grid spacing. Optimization was continued until the maximum forces on atoms were below 0.06 eV/Å. Final multifunctional model structures were constructed on the optimized Au_144_(*p*-MBA)_60_ structure by replacing some of the original *p*-MBA ligands with the optimized PEG-drug and PEG-peptide molecules. Exchange sites were selected randomly with a restriction of replacing only one ligand per single protecting RS-Au-SR unit. At the first step, a PEG-drug or a PEG-peptide molecule was added into the same bonding direction as the original *p*-MBA ligand. In the second phase, spatially free orientations of the PEG-drug or the PEG-peptide molecule were searched by rotational sampling around the S–C bond closest to the binding site. In the third phase, similar rotational sampling was done with respect to the S–Au bond of the protecting unit related to the binding site. The second phase sampling was repeated for each trial bonding angle of the third phase. Rotational sampling was done in steps of 3.6 degrees for the C–S bond and in steps of 10 degrees for the S–Au bond. The main goal was to avoid short nonphysical distances between the atoms, for which a minimum criterion of 1.75 Å was applied for all atomic distances, including the original Au atoms and *p*-MBA ligands and the already added PEG-drug and PEG-peptide molecules. All PEG-peptide molecules were added before the PEG-drug molecules. The described procedure favors first those spatially open binding orientations that are closer to the natural bonding direction because the sampling was terminated for each exchanged ligand immediately when the mentioned minimum criterion was fulfilled. Finally, the algorithm was run as many times as needed to fulfill the minimum distance criterion for all replaced ligands. For a better fitting, the PEG-drug and PEG-peptide ligands were added in elongated conformations that maximize the outreach from the cluster surface and optimize the free space around the exchanged molecules.

### Molecular Dynamics Simulations

The functionalized nanosystems were relaxed and simulated using GROMACS 2020^80^ MD simulation package with an Amber99sb-ildn force field for thiolate-protected nanoclusters,^69^ including the new parameters obtained for the different linker-peptide and linker-drug conjugates. All systems were simulated in a cubic box solvated with TIP3P water,^81^ with all *p*-MBA groups deprotonated.^82^ Sodium and chloride ions (0.15 M NaCl) were added to neutralize the systems. The SETTLE algorithm was used to constrain the internal degrees of freedom of the water molecules.^83^ Energy minimizations were carried out by using the steepest descent algorithm, followed by a short equilibration consisting of 10 ns NVT ensemble at 200 K and 10 ns NPT at 298.15 K and 1 bar pressure with position restraints on the heavy atoms of the nanoclusters. Afterward, 500 ns of production simulations was performed for each system, removing all the position restraints. Periodic boundary conditions (PBC), a leapfrog Verlet integrator with a 1 fs time step, a velocity-rescale thermostat with a reference temperature of 298.15 K and a coupling time constant of 0.1 ps,^84^ a 1.0 nm Lennard–Jones cutoff with dispersion correction for energy and pressure, particle-mesh Ewald (PME) method with a 1.0 nm cutoff and 0.12 nm grid spacing,^85^ and Berendsen barostat with a reference pressure of 1 bar and a coupling time constant of 1 ps^86^ were used. The lengths of covalent bonds containing hydrogens were constrained with the LINCS algorithm^87^ for improved performance.

All trajectories were visualized in VMD^88^ and analyzed with *gmx* commands included in GROMACS and in-house tcl scripts.

## Supporting Information Available

Figures of predicted poses of peptide candidates on AVB3 binding site, 3D models of multifunctional gold nanoclusters and tables of binding energies, calculated average values of radius of gyration and solvent-accessible surface area, calculated average distance between the gold core and the different components in the ligand layer, and calculated octanol/water partition coefficient of PEG-drug conjugates (PDF)
