# Supplementary material for: Ligand Ratio Plays a Critical Role in the Design of Optimal Multifunctional Gold Nanoclusters for Targeted Gastric Cancer Therapy
Source: ACS Nanosci Au. 2021 Jul 16;1(1):47–60. doi: 10.1021/acsnanoscienceau.1c00008 (PMC10125177; doi:10.1021/acsnanoscienceau.1c00008)
Supplement: Supplementary file 1 — ng1c00008_si_001.pdf [file ng1c00008_si_001.pdf]

Supporting Information for:

Ligand Ratio Plays a Critical Role in the Design of Optimal Multifunctional Gold Nanoclusters for Targeted Gastric Cancer Therapy

María Francisca Matus,† Sami Malola,† Hannu Häkkinen†,‡,*

†Department of Physics and ‡Department of Chemistry, Nanoscience Center, University of Jyväskylä, FI-40014 Jyväskylä, Finland.

*corresponding author. E-mail: hannu.j.hakkinen@jyu.fi

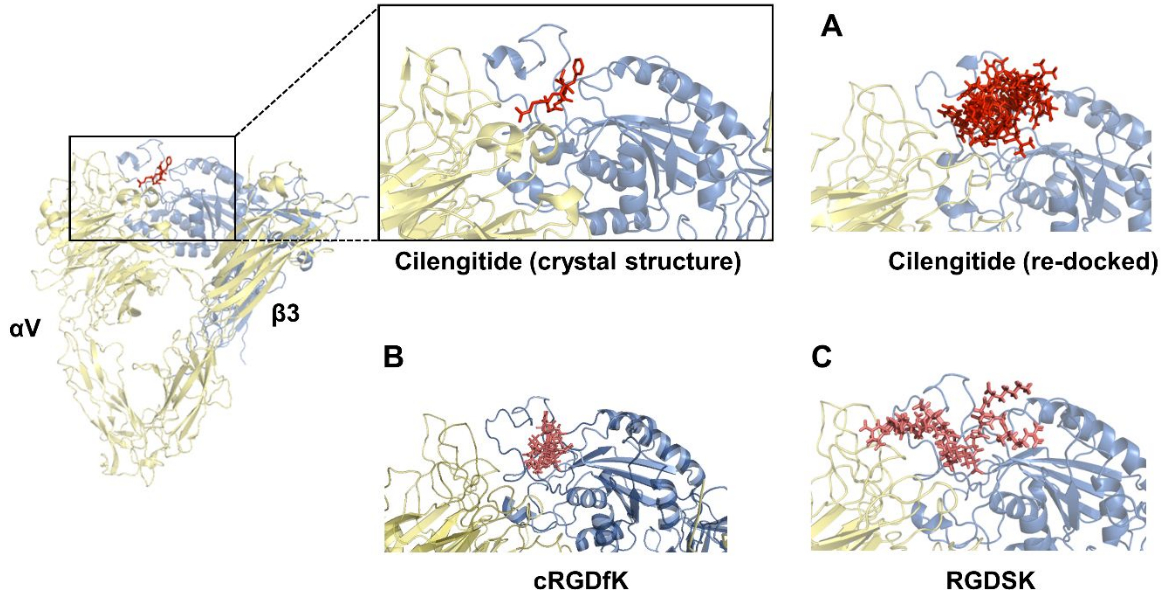

Figure S1. Pose of different peptides candidates in the αVβ3 binding site. Superimposition of the ten lowest energy poses obtained for (A) Cilengitide (re-docked), (B) cRGDfK and (C) RGDSK peptide. Cilengitide poses: red sticks; cRGDfK poses: pink sticks; RGDSK poses: pink sticks; αV subunit: yellow cartoon; β3 subunit: blue cartoon.

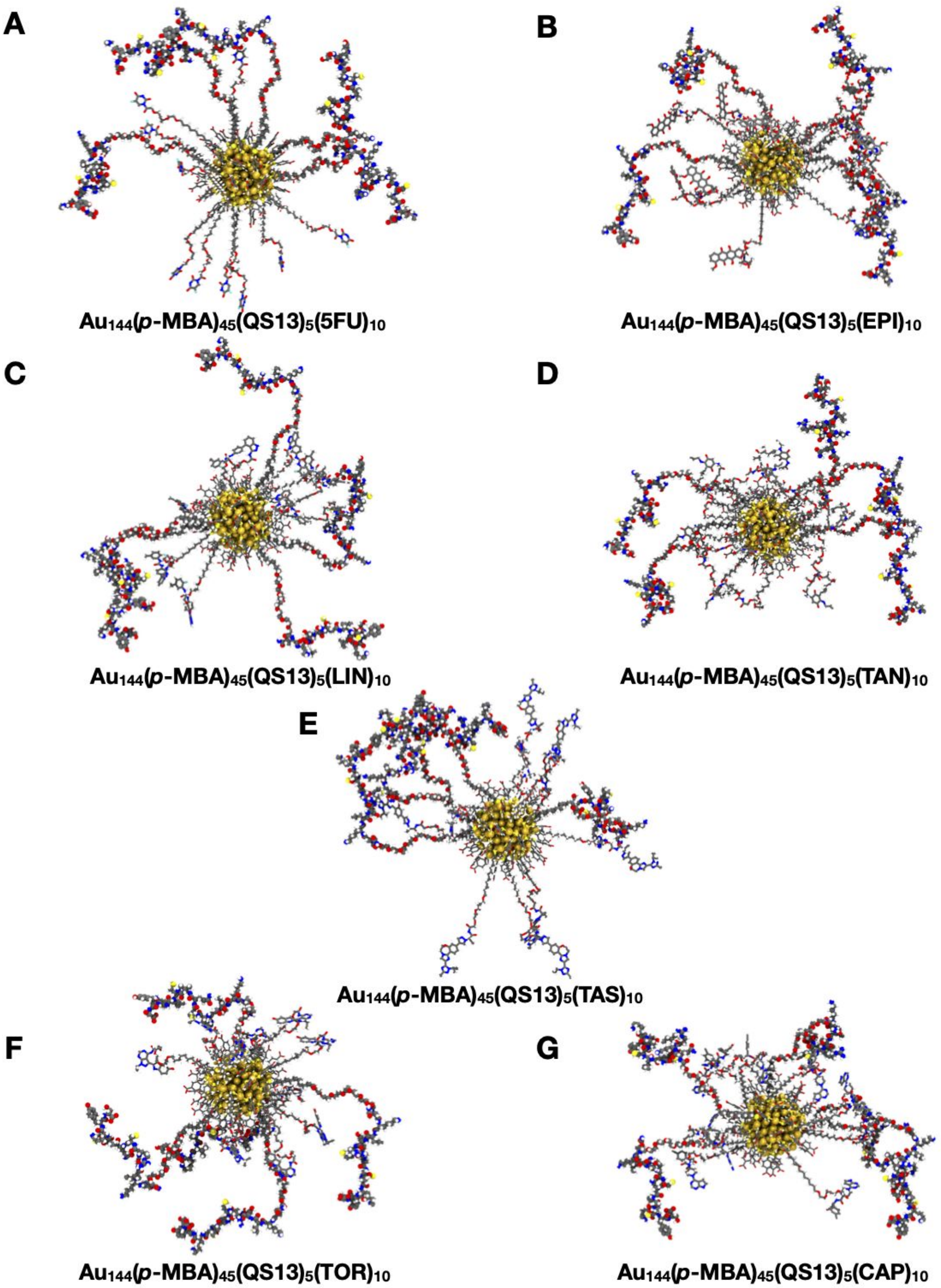

Figure S2. 3D models of multifunctional Au_{144} nanoclusters using QS13 peptide as targeting ligand and peptide:drug ratio 1:2. Model structures of Au_{144} nanoclusters functionalized with (A) 5-fluorouracil (5FU) (B) Epirubicin (EPI), (C) Linifanib (LIN), (D) Tanespimycin (TAN), (E) Taselisib (TAS), (F) Torkinib (TOR), or (G) Capivasertib (CAP) used as the initial configurations for the molecular dynamics simulations. Gold core is depicted as spheres, drugs as sticks, and peptides as balls. Color code: gold, golden yellow; sulfur, yellow, carbon, gray; oxygen, red; nitrogen, blue; hydrogen, white; fluoride, pink; chloride, dark gray.

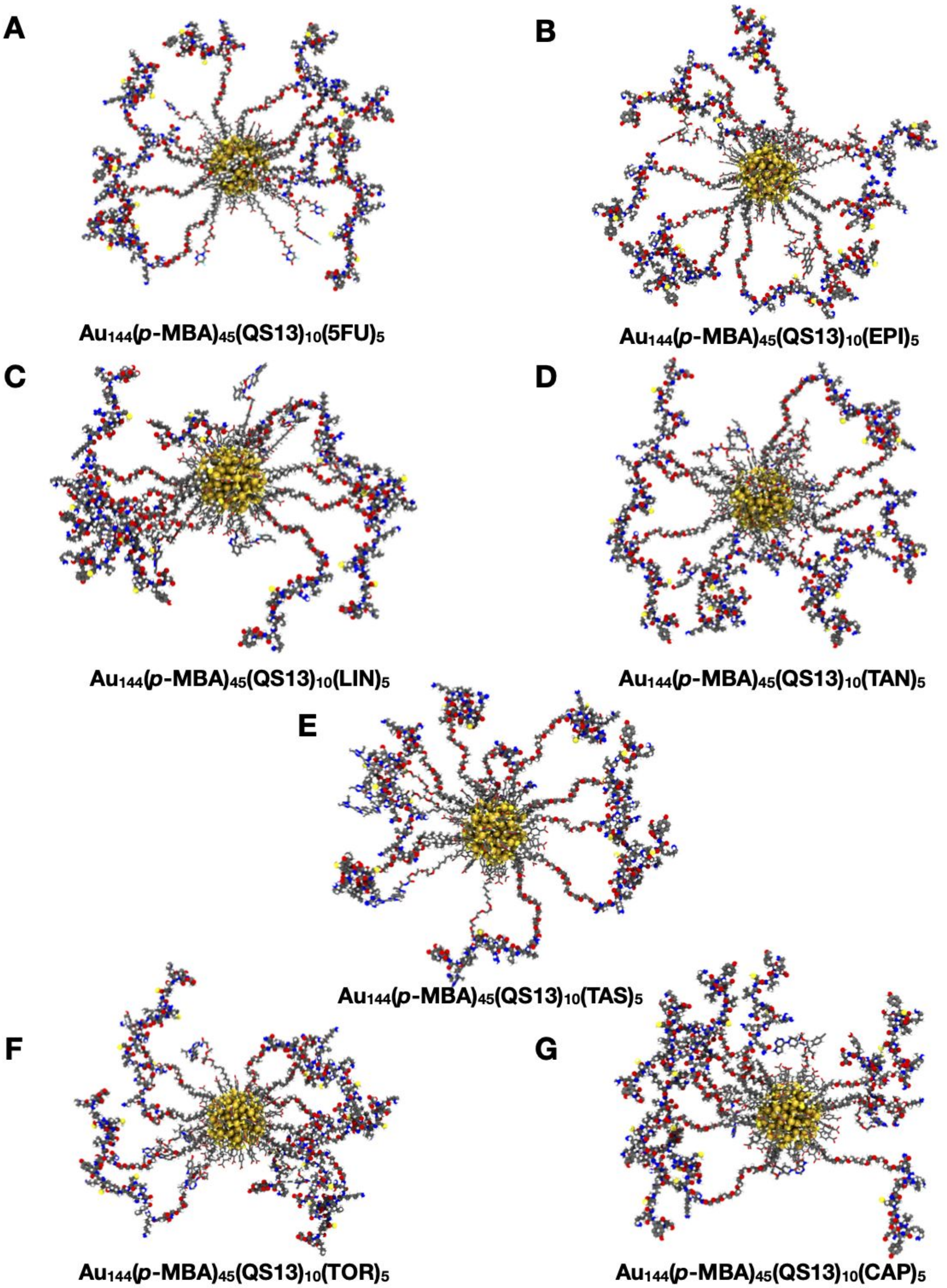

Figure S3. 3D models of multifunctional Au_{144} nanoclusters using QS13 peptide as targeting ligand and peptide:drug ratio 2:1. Model structures of Au_{144} nanoclusters functionalized with (A) 5-fluorouracil (5FU) (B) Epirubicin (EPI), (C) Linifanib (LIN), (D) Tanespimycin (TAN), (E) Taselisib (TAS), (F) Torkinib (TOR), or (G) Capivasertib (CAP) used as the initial configurations for the molecular dynamics simulations. Gold core is depicted as spheres, drugs as sticks, and peptides as balls. Color code: gold, golden yellow; sulfur, yellow, carbon, gray; oxygen, red; nitrogen, blue; hydrogen, white; fluoride, pink; chloride, dark gray.

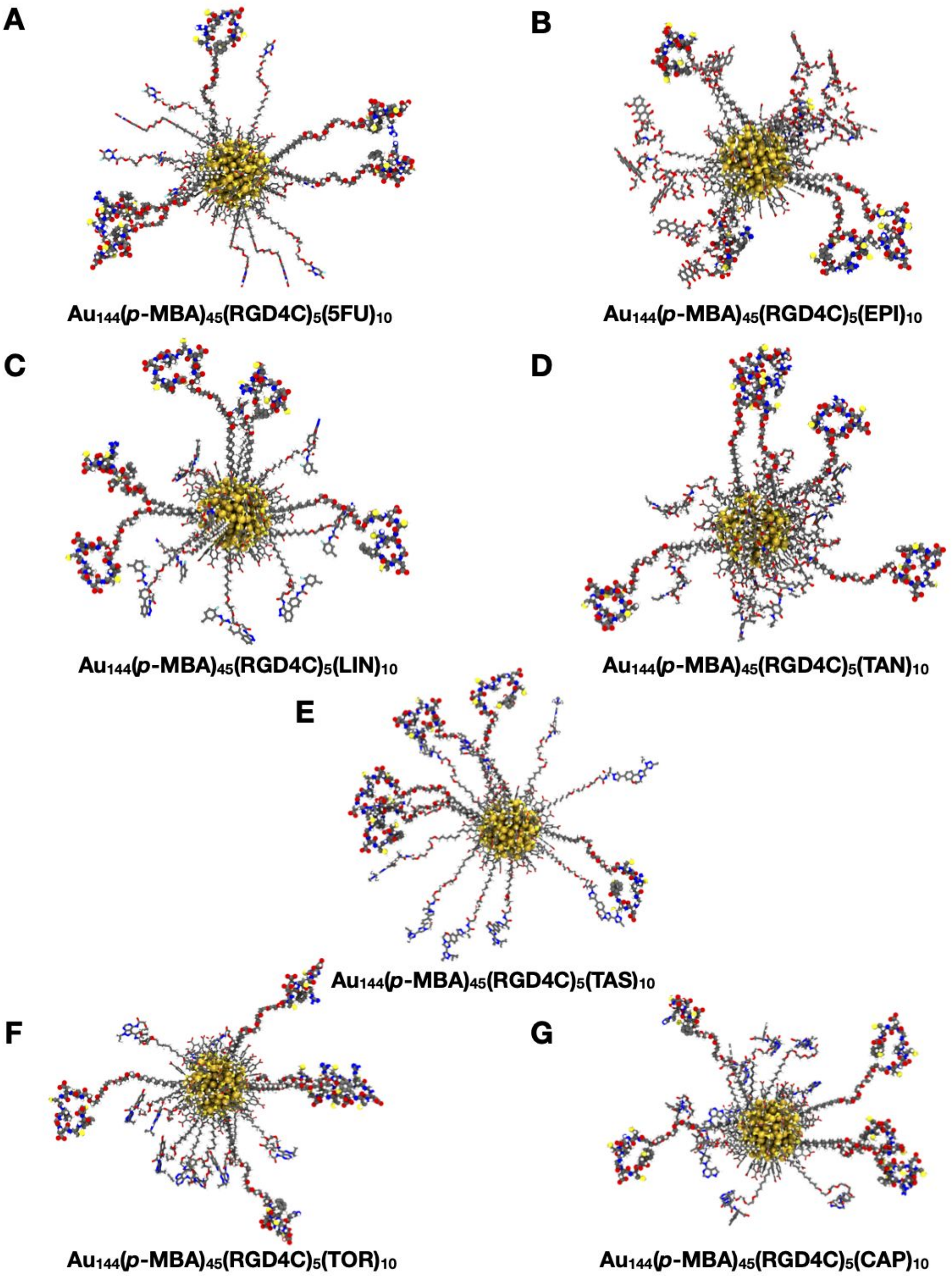

Figure S4. 3D models of multifunctional Au_{144} nanoclusters using RGD4C peptide as targeting ligand and peptide:drug ratio 1:2. Model structures of Au_{144} nanoclusters functionalized with (A) 5-fluorouracil (5FU) (B) Epirubicin (EPI), (C) Linifanib (LIN), (D) Tanespimycin (TAN), (E) Taselisib (TAS), (F) Torkinib (TOR), or (G) Capivasertib (CAP) used as the initial configurations for the molecular dynamics simulations. Gold core is depicted as spheres, drugs as sticks, and peptides as balls. Color code: gold, golden yellow; sulfur, yellow, carbon, gray; oxygen, red; nitrogen, blue; hydrogen, white; fluoride, pink; chloride, dark gray.

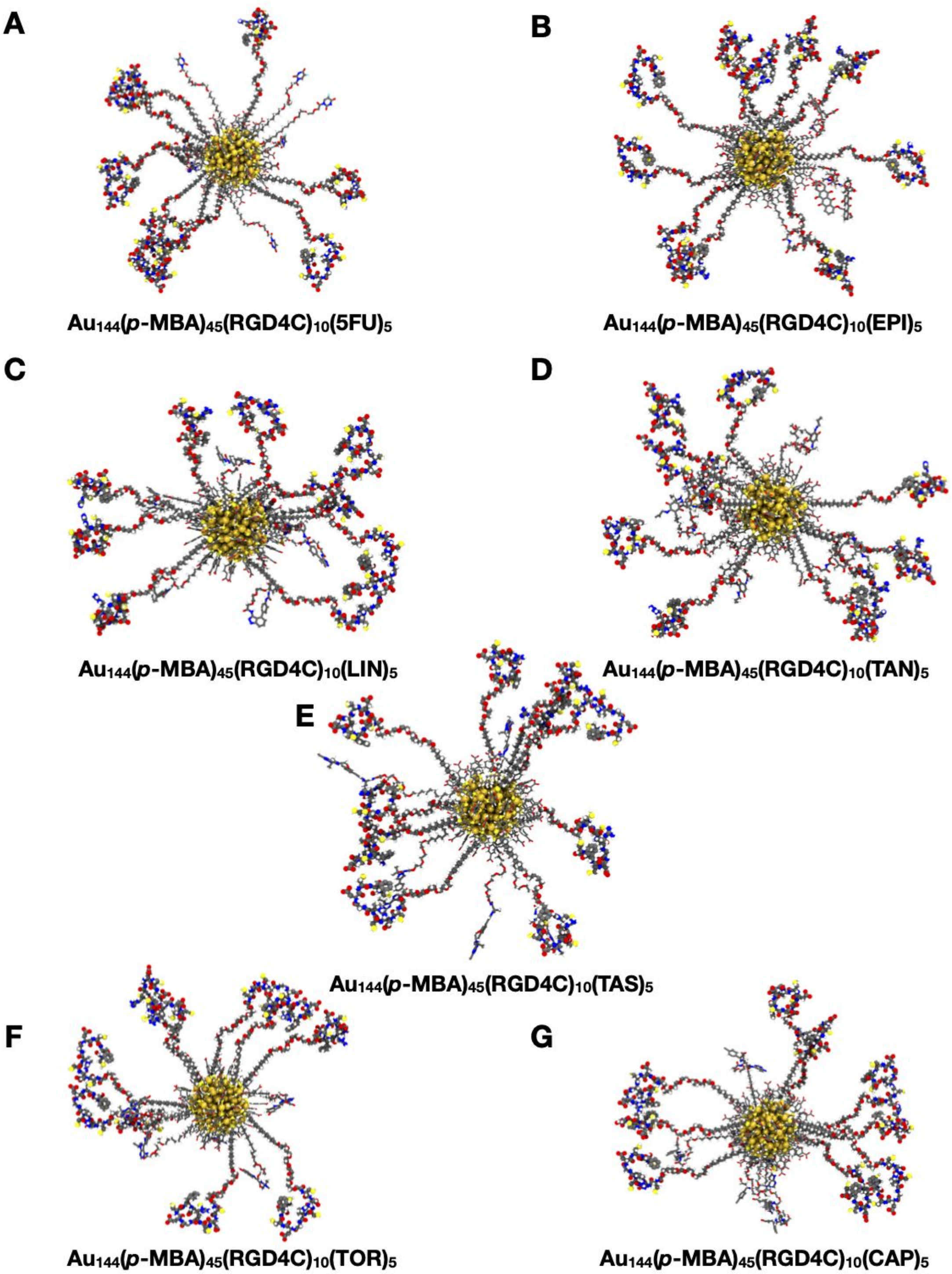

Figure S5. 3D models of multifunctional Au_{144} nanoclusters using RGD4C peptide as targeting ligand and peptide:drug ratio 2:1. Model structures of Au_{144} nanoclusters functionalized with (A) 5-fluorouracil (5FU) (B) Epirubicin (EPI), (C) Linifanib (LIN), (D) Tanespimycin (TAN), (E) Taselisib (TAS), (F) Torkinib (TOR), or (G) Capivasertib (CAP) used as the initial configurations for the molecular dynamics simulations. Gold core is depicted as spheres, drugs as sticks, and peptides as balls. Color code: gold, golden yellow; sulfur, yellow, carbon, gray; oxygen, red; nitrogen, blue; hydrogen, white; fluoride, pink; chloride, dark gray.

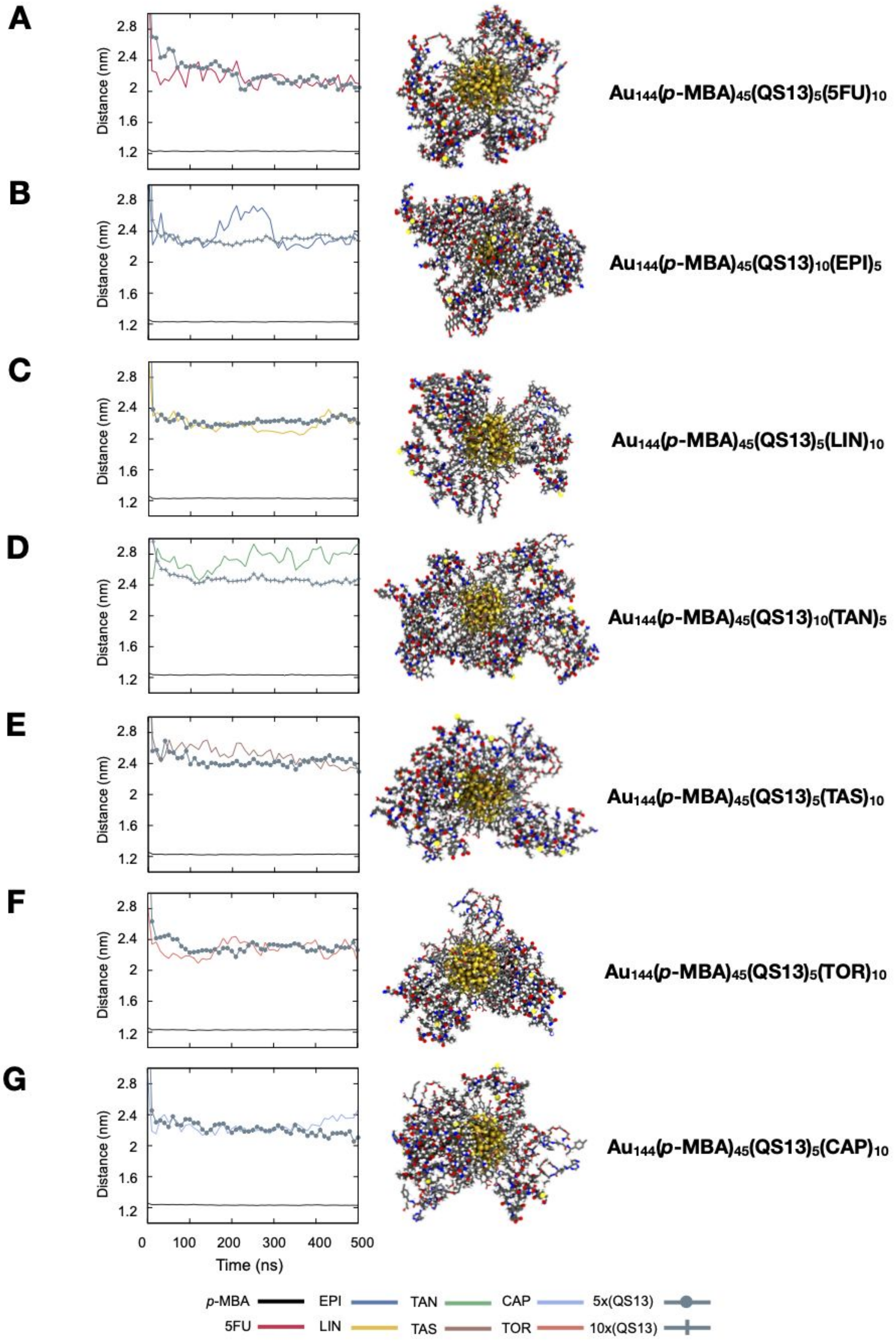

Figure S6. Distribution of the RCQVC motif of QS13 peptide on the ligand layer of less favorable formulations. Average distance between the gold core and the different components in the ligand layer (*para*-mercaptobenzoic acid, drug, and targeting motif of peptide) when the nanocluster is conjugated with (A) 5-

fluorouracil (5FU) (B) Epirubicin (EPI), (C) Linifanib (LIN), (D) Tanespimycin (TAN), (E) Taselisib (TAS), (F) Torkinib (TOR), or (G) Capivasertib (CAP). Right panels show a snapshot of the whole nanosystem after 500 ns of simulation. Gold core is depicted as spheres, drugs as sticks, and peptides as balls. Color code: gold, golden yellow; sulfur, yellow, carbon, gray; oxygen, red; nitrogen, blue; hydrogen, white; fluoride, cyan; chloride, dark gray.

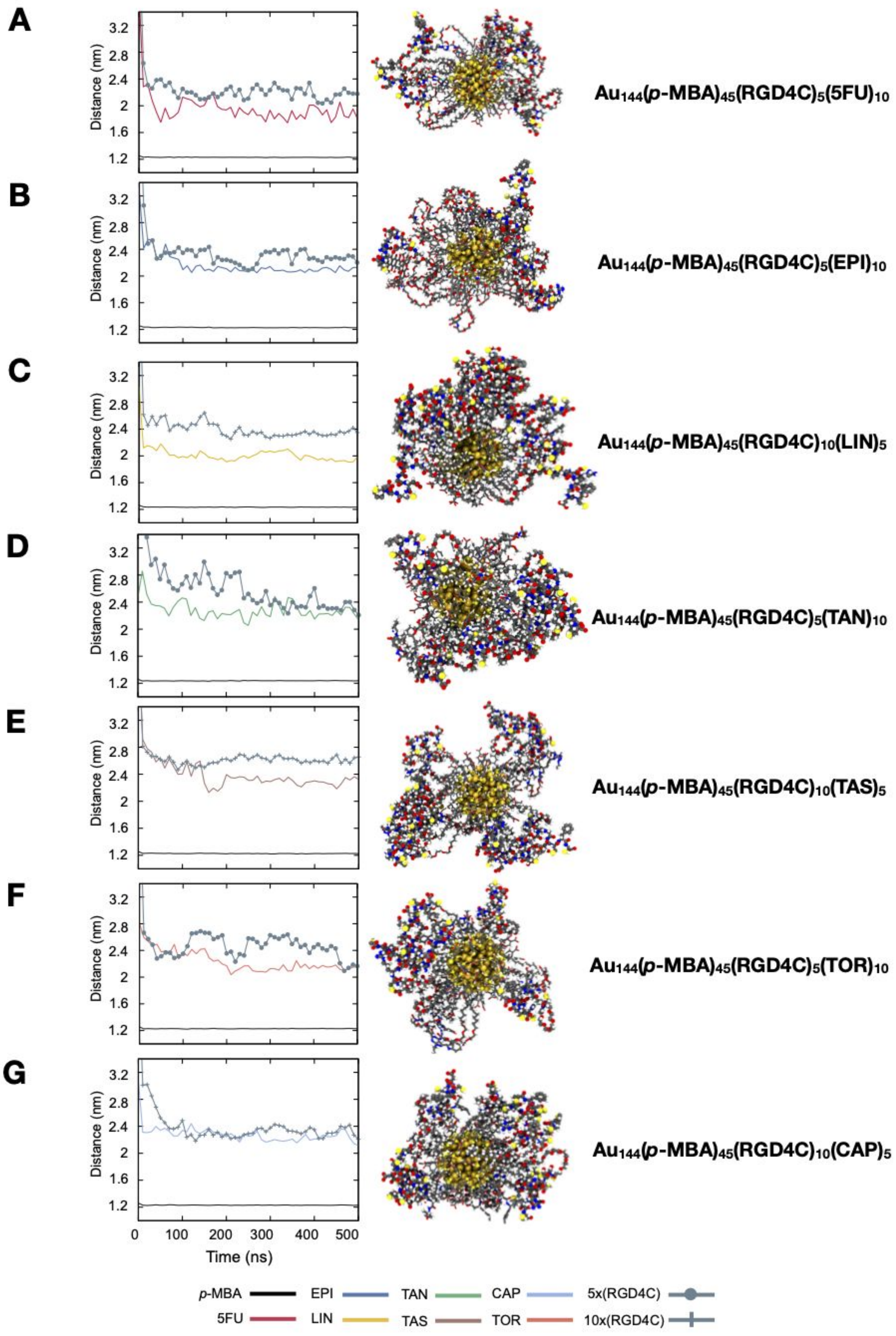

Figure S7. Distribution of the RGD motif of RGD4C peptide on the ligand layer of less favorable formulations. Average distance between the gold core and the different components in the ligand layer (*para*-mercaptobenzoic acid, drug, and targeting motif of peptide) when the nanocluster is conjugated with (A) 5-

fluorouracil (5FU) (B) Epirubicin (EPI), (C) Linifanib (LIN), (D) Tanespimycin (TAN), (E) Taselisib (TAS), (F) Torkinib (TOR), or (G) Capivasertib (CAP). Right panels show a snapshot of the whole nanosystem after 500 ns of simulation. Gold core is depicted as spheres, drugs as sticks, and peptides as balls. Color code: gold, golden yellow; sulfur, yellow, carbon, gray; oxygen, red; nitrogen, blue; hydrogen, white; fluoride, cyan; chloride, dark gray.

Table S1. Binding energy across the peptide–protein interface for the ten lowest energy conformations of RGD peptides.

Measurement	Peptide				
	**Cilengitide**	**QS13**	**RGD4C**	**RGDSK**	**cRGDfK**
Binding Energy (REU)	-12.28	-21.48	-19.85	-20.17	-7.85
	-14.27	-32.11	-13.19	-17.08	-8.02
	-12.90	-27.89	-13.17	-12.79	-8.16
	-11.59	-38.44	-24.42	-12.81	-13.37
	-17.78	-24.76	-23.81	-17.07	-13.39
	-14.14	-32.90	-26.92	-13.66	-13.91
	-17.36	-24.59	-22.47	-18.49	-10.52
	-15.23	-42.19	-18.59	-12.95	-13.38
	-14.59	-40.31	-17.25	-12.79	-13.30
	-15.15	-38.64	-23.33	-18.79	-11.67
Average	-14.53 ± 2.0	-32.33 ± 7.4	-20.30 ± 4.7	-15.66 ± 2.9	-11.36 ± 2.5

Binding energy (dG_separated) calculated for the 10 lowest energy conformations based on docking score from best 1,000 conformers. The crystal structure of Cilengitide (PDB ID: 1L5G)[1] was re-docked. REU: Rosetta energy units.

Table S2. Calculated average values of Radius of gyration (Rg) and Solvent-Accessible Surface Area (SASA) of QS13–functionalized Au_{144} nanoclusters.

System		Rg (nm)	SASA (nm^2)			
			Total	*p*-MBA	Drug	Peptide
Au_{144}(*p*-MBA)$_{60}$		1.17 ± 0.002	56.85 ± 0.73	56.83 ± 0.73	-	-
Functionalized Au_{144} NCs						
5x(QS13)	10x(5FU)	1.87 ± 0.05	135.69 ± 5.55	28.52 ± 1.66	32.19 ± 2.28	74.95 ± 4.14
	10x(EPI)	2.07 ± 0.05	154.14 ± 4.84	29.60 ± 1.25	55.85 ± 1.89	68.68 ± 3.35
	10x(LIN)	1.93 ± 0.03	138.58 ± 4.79	26.51 ± 1.49	36.18 ± 1.93	75.87 ± 3.09
	10x(TAN)	2.01 ± 0.04	159.02 ± 5.90	27.70 ± 1.86	66.13 ± 3.79	65.19 ± 3.38
	10x(TAS)	2.02 ± 0.04	158.06 ± 7.32	28.41 ± 1.44	58.12 ± 3.84	71.52 ± 4.26
	10x(CAP)	1.94 ± 0.04	149.02 ± 6.32	25.37 ± 1.52	49.58 ± 2.59	74.06 ± 5.07
	10x(TOR)	1.89 ± 0.04	142.03 ± 6.25	28.03 ± 1.79	45.10 ± 2.23	68.89 ± 3.83
10x(QS13)	5x(5FU)	2.02 ± 0.04	177.50 ± 6.83	21.20 ± 1.42	16.66 ± 1.81	139.63 ± 6.03
	5x(EPI)	2.07 ± 0.02	183.86 ± 8.31	19.89 ± 1.83	28.28 ± 3.04	135.68 ± 5.78
	5x(LIN)	2.00 ± 0.03	167.69 ± 6.22	23.91 ± 1.58	21.12 ± 1.95	122.65 ± 4.83
	5x(TAN)	2.23 ± 0.03	189.17 ± 6.56	24.13 ± 1.55	32.80 ± 2.83	132.23 ± 4.69
	5x(TAS)	2.05 ± 0.06	179.37 ± 11.11	22.19 ± 2.85	25.34 ± 2.37	131.83 ± 6.96
	5x(CAP)	2.07 ± 0.04	182.17 ± 7.19	21.31 ± 2.03	20.89 ± 1.55	139.97 ± 5.29
	5x(TOR)	2.07 ± 0.06	173.80 ± 10.44	27.74 ± 1.59	17.05 ± 1.61	131.98 ± 8.32

The values were averaged between 30 – 500 ns simulated time.

p-MBA: *para*-mercaptobenzoic acid; 5FU: 5-fluorouracil; EPI: Epirubicin; LIN: Linifanib; TAN: Tanespimycin; TAS: Taselisib; CAP: Capivasertib; TOR: Torkinib.

Table S3. Calculated average values of Radius of gyration (Rg) and Solvent-Accessible Surface Area (SASA) of RGD4C–functionalized Au_{144} nanoclusters.

System		Rg (nm)	SASA (nm^2) Total	*p*-MBA	Drug	Peptide
Au_{144}(*p*-MBA)$_{60}$		1.17 ± 0.002	56.85 ± 0.73	56.83 ± 0.73	-	-
Functionalized Au_{144} NCs						
5x(RGD4C)	10x(5FU)	1.83 ± 0.04	124.10 ± 5.89	30.80 ± 1.36	35.74 ± 2.61	57.54 ± 3.83
	10x(EPI)	1.92 ± 0.06	145.19 ± 6.63	28.68 ± 2.49	62.72 ± 2.96	53.78 ± 2.85
	10x(LIN)	2.01 ± 0.04	132.55 ± 3.85	31.68 ± 1.13	43.16 ± 2.14	57.71 ± 2.87
	10x(TAN)	1.95 ± 0.08	153.77 ± 7.79	28.85 ± 2.50	67.67 ± 3.39	57.25 ± 4.42
	10x(TAS)	2.01 ± 0.06	137.45 ± 6.35	33.45 ± 1.11	51.83 ± 5.11	52.16 ± 2.50
	10x(CAP)	1.96 ± 0.06	139.28 ± 6.86	31.94 ± 1.54	47.64 ± 4.12	59.68 ± 2.89
	10x(TOR)	1.89 ± 0.07	128.55 ± 5.03	30.74 ± 2.29	39.10 ± 2.50	58.70 ± 3.33
10x(RGD4C)	5x(5FU)	1.99 ± 0.07	150.52 ± 7.44	30.51 ± 1.50	12.99 ± 1.92	107.00 ± 5.83
	5x(EPI)	2.05 ± 0.06	165.84 ± 9.31	25.68 ± 2.71	28.44 ± 3.13	111.71 ± 5.67
	5x(LIN)	1.92 ± 0.05	148.76 ± 7.71	25.38 ± 1.27	18.00 ± 1.85	105.37 ± 6.12
	5x(TAN)	2.17 ± 0.05	164.32 ± 7.93	26.84 ± 1.43	26.07 ± 2.76	111.40 ± 5.24
	5x(TAS)	2.14 ± 0.04	166.53 ± 6.39	29.52 ± 1.49	27.53 ± 2.00	109.46 ± 5.08
	5x(CAP)	2.01 ± 0.06	158.24 ± 1.89	26.04 ± 1.72	23.94 ± 1.89	108.26 ± 5.36
	5x(TOR)	2.09 ± 0.08	160.51 ± 10.00	31.73 ± 1.92	21.56 ± 1.65	107.21 ± 8.02

The values were averaged between 30 – 500 ns simulated time.

p-MBA: *para*-mercaptobenzoic acid; 5FU: 5-fluorouracil; EPI: Epirubicin; LIN: Linifanib; TAN: Tanespimycin; TAS: Taselisib; CAP: Capivasertib; TOR: Torkinib.

Table S4. Calculated average distance between the gold core and the different components in the ligand layer (*p*-MBA, drug, and targeting motif of peptide) of QS13–functionalized Au_{144} nanoclusters.

System		Distance (nm)		
		***p*-MBA–gold core**	**Drug–gold core**	**RCQVC motif–gold core**
$Au_{144}(p\text{-MBA})_{60}$		1.23 ± 0.000	-	-
Functionalized Au_{144} NCs				
5x(QS13)	10x(5FU)	1.23 ± 0.002	2.17 ± 0.09	2.21 ± 0.13
	10x(EPI)	1.23 ± 0.002	2.45 ± 0.06	2.45 ± 0.09
	10x(LIN)	1.23 ± 0.002	2.19 ± 0.09	2.23 ± 0.04
	10x(TAN)	1.23 ± 0.003	2.38 ± 0.06	2.37 ± 0.10
	10x(TAS)	1.23 ± 0.003	2.50 ± 0.11	2.43 ± 0.07
	10x(CAP)	1.23 ± 0.004	2.26 ± 0.08	2.21 ± 0.07
	10x(TOR)	1.23 ± 0.003	2.27 ± 0.09	2.30 ± 0.06
10x(QS13)	5x(5FU)	1.23 ± 0.002	2.15 ± 0.12	2.25 ± 0.06
	5x(EPI)	1.23 ± 0.002	2.36 ± 0.17	2.23 ± 0.04
	5x(LIN)	1.23 ± 0.003	2.17 ± 0.10	2.28 ± 0.06
	5x(TAN)	1.23 ± 0.003	2.73 ± 0.11	2.47 ± 0.04
	5x(TAS)	1.23 ± 0.002	2.27 ± 0.08	2.44 ± 0.10
	5x(CAP)	1.23 ± 0.002	2.37 ± 0.08	2.35 ± 0.06
	5x(TOR)	1.23 ± 0.002	2.04 ± 0.05	2.33 ± 0.08

The values were averaged between 30 – 500 ns simulated time and based on the center of mass of each component.

p-MBA: *para*-mercaptobenzoic acid; 5FU: 5-fluorouracil; EPI: Epirubicin; LIN: Linifanib; TAN: Tanespimycin; TAS: Taselisib; CAP: Capivasertib; TOR: Torkinib.

Table S5. Calculated average distance between the gold core and the different components in the ligand layer (*p*-MBA, drug, and targeting motif of peptide) of RGD4C–functionalized Au_{144} nanoclusters.

System		Distance (nm)		
		***p*-MBA–gold core**	**Drug–gold core**	**RGD motif–gold core**
$Au_{144}(p\text{-MBA})_{60}$		1.23 ± 0.000	-	-
Functionalized Au_{144} NCs				
5x(RGD4C)	10x(5FU)	1.23 ± 0.002	1.92 ± 0.11	2.20 ± 0.09
	10x(EPI)	1.23 ± 0.003	2.13 ± 0.10	2.30 ± 0.09
	10x(LIN)	1.23 ± 0.002	2.22 ± 0.08	2.80 ± 0.11
	10x(TAN)	1.23 ± 0.003	2.26 ± 0.10	2.54 ± 0.22
	10x(TAS)	1.23 ± 0.002	2.31 ± 0.10	3.07 ± 0.12
	10x(CAP)	1.23 ± 0.003	2.34 ± 0.09	2.48 ± 0.11
	10x(TOR)	1.23 ± 0.002	2.21 ± 0.13	2.45 ± 0.15
10x(RGD4C)	5x(5FU)	1.23 ± 0.002	1.99 ± 0.10	2.48 ± 0.09
	5x(EPI)	1.23 ± 0.003	1.98 ± 0.07	2.45 ± 0.12
	5x(LIN)	1.23 ± 0.002	2.00 ± 0.06	2.38 ± 0.10
	5x(TAN)	1.23 ± 0.002	2.22 ± 0.09	2.62 ± 0.10
	5x(TAS)	1.23 ± 0.003	2.35 ± 0.13	2.60 ± 0.05
	5x(CAP)	1.23 ± 0.002	2.26 ± 0.07	2.34 ± 0.12
	5x(TOR)	1.23 ± 0.002	2.28 ± 0.16	2.51 ± 0.14

The values were averaged between 30 – 500 ns simulated time and based on the center of mass of each component.

p-MBA: *para*-mercaptobenzoic acid; 5FU: 5-fluorouracil; EPI: Epirubicin; LIN: Linifanib; TAN: Tanespimycin; TAS: Taselisib; CAP: Capivasertib; TOR: Torkinib.

Table S6. Calculated octanol/water partition coefficient (logP) of PEG-drug conjugates used in the functionalization of Au_{144} nanoclusters using ALOGPS 2.1 software package.[2]

Conjugate	logP
PEG-5FU	8.44
PEG-EPI	7.83
PEG-LIN	9.17
PEG-TAN	8.86
PEG-TAS	9.17
PEG-CAP	9.04
PEG-TOR	9.71

PEG: polyethylene glycol; 5FU: 5-fluorouracil; EPI: Epirubicin; LIN: Linifanib; TAN: Tanespimycin; TAS: Taselisib; CAP: Capivasertib; TOR: Torkinib.

References

(1) Xiong, J.-P.; Stehle, T.; Zhang, R.; Joachimiak, A.; Frech, M.; Goodman, S. L.; Arnaout, M. A. Crystal Structure of the Extracellular Segment of Integrin αVβ3 in Complex with an Arg-Gly-Asp Ligand. *Science.* **2002**, *296* (5565), 151–155.

(2) Tetko, I. V; Gasteiger, J.; Todeschini, R.; Mauri, A.; Livingstone, D.; Ertl, P.; Palyulin, V. A.; Radchenko, E. V; Zefirov, N. S.; Makarenko, A. S. Virtual Computational Chemistry Laboratory–design and Description. *J. Comput. Aided. Mol. Des.* **2005**, *19* (6), 453–463.
